# Paving the Way for Outdoor Play: Examining Socio-Environmental Barriers to Community-Based Outdoor Play

**DOI:** 10.3390/ijerph18073617

**Published:** 2021-03-31

**Authors:** Janet Loebach, Marcos Sanches, Julia Jaffe, Tara Elton-Marshall

**Affiliations:** 1 Department of Design + Environmental Analysis, Cornell University, Ithaca, New York, NY 14853, USA; jgj57@cornell.edu; 2 Krembli Centre for Neuroinformatics, Centre for Addiction and Mental Health, London, ON N6G 4X8, Canada; marcos.sanches@camh.ca; 3 Centre for Addiction and Mental, Institute for Mental Health Policy Research, London, ON N6G 4X8, Canada; tara.eltonmarshall@camh.ca; 4 Campbell Family Mental Health Research Institute, Toronto, ON M5S 2S1, Canada; 5 Dalla Lana School of Public Health, Toronto, ON M5T 3M7, Canada; 6 Department of Epidemiology and Biostatistics, Western University, London, ON N6A 5C1, Canada

**Keywords:** outdoor play, time outdoors, independent mobility, community environments, neighborhood, barriers, screen-based activity, community planning, child-friendly communities

## Abstract

Outdoor play and independent, neighborhood activity, both linked with healthy childhood development, have declined dramatically among Western children in recent decades. This study examines how social, cultural and environmental factors may be hindering children’s outdoor and community-based play. A comprehensive survey was completed by 826 children (aged 10–13 years) and their parents from 12 schools (four each urban, suburban and rural) from a large county in Ontario, Canada. Five multilevel regression models, controlling for any school clustering effect, examined associations between outdoor play time per week and variable sets representing five prevalent factors cited in the literature as influencing children’s outdoor play (OP). Models predicted that younger children and boys were more likely to spend time playing outdoors; involvement in organized physical activities, other children nearby to play with, higher perception of benefits of outdoor play, and higher parental perceptions of neighborhood social cohesion also predicted more time in outdoor play. Time outdoors was less likely among children not allowed to play beyond home without supervision, felt they were ‘too busy’ with screen-based activities, and who reported higher fears related to playing outdoors. Study findings have important implications for targeting environmental, cultural and policy changes to foster child-friendly communities which effectively support healthy outdoor play.

## 1. Introduction

Engagement in unsupervised outdoor activities, such as ‘free’ or ‘unstructured’ play and independent community-based activity, has been shown to be as a primary facilitator of cognitive, social, physical, and psychological development in young people, and linked to key youth health and wellbeing outcomes [1,2,3,4,5,6]. ‘Outdoor environments that afford diverse opportunities for unstructured play can help stimulate children’s creativity and problem-solving abilities, improve learning outcomes, and support healthy physical, social, and emotional development [7,8,9,10,11]. Outdoor, free and nature-rich play also supports healthy brain development by encouraging exploration and building activities, which in turn can strengthen wayfinding, orientation, and decision making skills, and the ability to respond to changing contexts [9,12,13,14,15].

However, there is a growing, global trend that the time young people in Western countries spend outdoors has declined dramatically in recent decades [15,16,17]. Evidence illustrates that contemporary Western youth are playing less frequently outdoors [15,18,19,20,21], are less likely to independently travel through their neighborhoods, especially during their leisure time [22,23,24] and are getting less daily physical activity outdoors than previous generations [11,17,25]. Children are also more inclined to engage in sedentary pursuits indoors at home, such as screen-based or digital media activities, than their parents or grandparents [21,25,26]. According to the 2012–2013 Canadian Health Measures survey [27], Canadian children and adolescents are spending more than half of their waking hours engaged in sedentary behaviors [11,28,29]. The decrease in time youth spend in active outdoor play combined with the marked increase in daily sedentary activities are contributing to a number of negative physical and psychosocial health outcomes among children and youth—such as increased rates of obesity, diabetes, lower self-esteem, and fewer pro-social behaviors [1,11,15,30,31,32].

Recent work suggests that increased time outdoors may help to facilitate healthy behaviors and combat or moderate negative health consequences for youth. A recent systematic review concluded that children between the ages of 3 and 12 who spend more time outdoors accumulate more physical activity and spend less time in sedentary activity [6]. Larouche et al. [28] found that for 7-to-14-year-olds, each additional hour spent outdoors daily was associated with an additional seven minutes of moderate-to-vigorous physical activity (MVPA) and a 13-min decrease in sedentary activity [28]. As playing outdoors is a natural way for youth to be physically active in their daily lives, declines in outdoor play may be one factor contributing to lower physical activity and increased health concerns in today’s youth [33,34].

Children’s ability to spend time playing outdoors at home and around their communities may be tied to the level of independent mobility (IM) they are granted by parents and guardians, that is, “children’s freedom to move around their neighborhoods and cities on their own, without adults” [30] (p. 2). As children age and mature, parents’ perceptions of their child’s competence and ability to navigate challenging situations on their own out in their neighborhood typically increases, and they subsequently award their children greater freedom and autonomy to spend time beyond home and mobility to travel longer distances independently [35,36,37,38,39,40]. The ability of children to travel around and spend time independently in their neighborhood environment has important positive implications for building social skills and capital [41,42], emotional development [17,43], cognitive development [24] and a significant positive effect on health outcomes including bone health, healthy weight, and protection against future chronic diseases [4,44,45,46] Greater mobility also affords youth increased opportunities to interact with peers, develop decision-making and risk-assessment skills, construct their self-identities, and gain the competence and confidence to successfully and safely navigate their neighborhoods [30,47,48].

However, like levels of outdoor play, studies from many Western countries have highlighted a similar decline in children’s IM in their neighborhoods—both in terms of time spent being independently mobile and the extent to which children have the freedom and range to explore their localities [17,22,43]. Restricting independent mobility has been linked with reduced physical activity among children [49,50], decreases in opportunities to learn about their neighborhoods, and negative impacts on the development of spatial skills [24,51]. The causes of these declines in both time outdoors and neighborhood mobility are multiple and interwoven and have been linked to numerous elements of the social, cultural, and physical environment.

### 1.1. Factors Influencing Time Outdoors and Independent Mobility

Recent reviews and meta-analyses have identified contributing factors to the decline in outdoor time and independent mobility at different levels of the socio-ecological model [52], ranging from the child (age, gender/sex) and family level (family structure, socioeconomic status (SES), ethnicity, level of support for time outdoors and IM), to the neighborhood environment (urbanization, walkability, prevalence of destinations and amenities) to societal and cultural attitudes (social norms, neighborhood perceptions) [36,39,43,53]. Despite some conflicting findings, numerous studies have found that demographics such as age, gender, ethnicity, and socio-economic status, all moderate children’s access to the outdoors for leisure and play as well as their independent mobility [33,36,54,55,56]. The urbanicity of the home environment may also be influential; two recent Canadian studies found higher levels of outdoor play among children in rural communities compared to their urban counterparts [57,58]. Delisle Nystrom et al. [57] found this association was stronger for girls. However, while some studies suggest that rural children have fewer parent-imposed restrictions on community-based activity [59], others have found that rural children can face more barriers to accessing community-based play spaces such as parks and playgrounds [37], and that proximity to community play spaces has positively influenced levels of outdoor play [36,60,61]. Others have linked urban neighborhoods with increased amenities for youth, greater neighborhood walkability [62], as well as higher levels of physical activity and less time in sedentary activities among youth [63]. In contrast, Aarts et al. [34] found that living in an urban city center was negatively associated with outdoor play for boys 7 to 9 years old; Bringolf-Isler et al. [64] found outdoor play was inversely associated with the street density inherent in more urbanized areas.

While evidence to date generally confirms that increasing age affords young people increased freedom and independent mobility, studies have also found that age is inversely associated with outdoor play and time outside [64,65]. Age has also been positively correlated with screen time and media usage [21,66], which has been associated with increased sedentary activities and less time spent outdoors [30,67]. Some studies have suggested that the decline in outdoor play seen in older children and adolescents may be associated with individual-level factors such as lower perceived enjoyment from outdoor play or social-level factors like peer support and peer perceptions of outdoor play [68,69]. Lack of outdoor time in older children has also been linked to increased school and academic engagements, increased screen device use, spending more time with peers indoors, and time mismanagement [64,69].

Findings related to connections between outdoor time/play gender are mixed. However, studies have reported that male children are generally granted more freedom in the type of play and distances they are allowed to travel due to increased parental fears over cultural expectations and safety fears for female children [53,69,70]. Female children have also described the presence of groups of older children in their neighborhood or play spaces as a barrier to engaging in play and outdoor activities [67]. Some scholars have concluded that factors such as the type of activity, destination and social companions are influencing outdoor activity and IM more than child gender [36,39,54].

### 1.2. The Impact of Neighborhood Perceptions and Conditions on Outdoor Play

Historically, the neighborhood environment has been a common setting for Western children’s time outdoors and outdoor play [38,51,71]. The time and motivation children have for outdoor activity in their neighborhood or community is influenced by perceptions of multiple social, cultural, and environmental factors, including the physical infrastructure, cultural norms, and social characteristics of the neighborhood [22,34,39,72]. Parents’ perception of neighborhood safety, the availability of spaces for play, the presence of other children, and a sense of neighborhood cohesion have all been shown to affect if and how children spend time outside in their communities [54,56,70,73,74,75,76]. The presence of other children within walking distance is an important facilitator of children’s unstructured, outdoor activity, as it affords impromptu opportunities for group play. It can also give parents a sense of collective safety, which can prompt them to give their children greater freedom when they are outside with friends [54,72].

Neighborhood safety issues are one of the most commonly reported sources of parental concern and a primary reason for limiting children’s time outdoors and IM [37,54,56,70,77]. Parents have reported fears of accidents or injury [37,77,78,79,80], strangers [78,79], traffic [23,30,54], and antisocial behavior or bullying from other youth [78,80]. However, it has also been posited that parental restrictions of children’s independent mobility and time outside is, “likely related to parents’ separation anxiety more generally and influenced by their personality, particularly their attachment style” [77] (p. 2254). Parental concerns over injury and risk during play have shifted many of the activities in which children are allowed to engage from unstructured, outdoor play to more structured and indoor pursuits, including adult-supervised play and academic activities [22,38,75,78,81,82]. This culture of fear of risk has not only led to an underestimation of children’s abilities, but also to a reduction in opportunities for children to gain competence in judging risky situations and the ability to negotiate future risks independently [54,83,84,85,86].

The neighborhood social environment, especially community social cohesion, has emerged as an important influencer of parents’ perceptions of the neighborhood and of time children spend playing outdoors [70,87]. Social cohesion within the neighborhood is characterized by strong social bonds and relationships among parents and infrequent social conflicts [88,89]. These strong social bonds may facilitate children’s outdoor time and play through increased trust and communication among parents in the neighborhood [54,70,90]. Additionally, the cultural and social norms around the acceptability of children’s outdoor play greatly influences parenting practices within their neighborhood. Parents tend to follow implicit norms about the ‘right way’ to supervise children such that they are protected from danger and are not a bother to other residents [36,39,54].

Parents’ perception of neighborhood safety and attitudes towards spending time outside have a strong influence on their children’s developing views of their neighborhood [54,64,67,70,91]. A UK study with 8-to-9-year-olds found children to be less concerned with more ‘traditional’ adult fears of traffic and strangers than they were with perceived risks posed by other young people in their outdoor places [67]. However, Brussoni et al. [54] found that children internalize their parent’s perceptions of neighborhood safety—influencing both children’s self-imposed boundaries to outdoor activity and their interpretation and assessment of the people and places around their neighborhood.

Parental support for outdoor activities, including encouragement to play and incentives to exercise, and enrollment of children in sports or clubs, have all been shown to significantly influence physical activity and time spent outdoors [70,76,83,92,93]. Encouraging outdoor time and play also promotes the integration of these types of experiences in family routines and is positively associated with outdoor play for all ages of youth [34,76,83].

### 1.3. Time Available for Unstructured, Unsupervised Activities

Declines in outdoor play may also be a consequence of an overall reduction in free time available to children, or the growing appeal of indoor, screen-based activities. Recent studies have reported increased daily screen time, augmented electronic media use, and increasingly structured leisure time for Western children and youth [53,66]. In their 2019 study, Larson and colleagues [66] found that among a sample of youth aged 10–15 years, most individuals reported more cumulative screen and media time than time spent outdoors. These two activities were significantly and inversely related, pointing to augmented electronic media use as severely compromising the amount of time that youth spend playing outside. The growth and the availability of new technology enables children and youth to engage and communicate with friends through digital devices such as cellphones, tablets, computers, and gaming systems without ever leaving their homes [30,94]. This rapid rise of electronic media has been identified as a key factor in declining nature-based outdoor time for youth [66,95,96].

The after-school period has been highlighted as a critical opportunity for unstructured outdoor play for children [28,97]. However, many parents have come to view this period as an opportunity to direct their children towards additional academically oriented pursuits or other structured extracurricular activities [70,98]. Consequently, many Western children are more likely to spend this after-school time in more structured, supervised, or indoor activities during the after-school period [75,99], which may limit time available for free play outdoors. Beyond these more structured activities, the 2014–2016 Kids CANPLAY report highlighted that the majority of Canadian youth (5-to-19-year-olds) engage in indoor, sedentary activities after school such as doing homework (67%) or playing computer or video games, watching tv, and reading (77%) [100,101,102]. These changes in after-school activity patterns may be contributing to significant declines in the proportion of Canadian youth who play outdoors after school (86% of 5-to-10-year-olds and 34% of 15-to-19-year-olds) or participate in unorganized physical activities or sports (87% of 5-to-10-year-olds and 61% of 15-to-19-year-olds) [100,101,102].

### 1.4. Limitations of Outdoor Play Research to Date

The ways in which time spent outdoors in children has been studied to date varies widely in terms of participant age, methodology, and foci, confounding scholars’ ability to compare findings. Many studies also conflate outdoor play and physical activity, as well as children’s IM with active travel, which undermines the value of travel and outdoor play that is not active [30,103,104]. A majority of studies on these topics to date have also been carried out primarily in urban areas in the US, Canada, and Australia, and do not consider the potentially differing outdoor play patterns of children living in rural areas. Many studies on children’s IM and outdoor activities have also relied on parent-proxies rather child reports or objective measures [39,105]. Few studies to date have simultaneously investigated a diverse range of influential factors on children’s outdoor play or IM.

The time that children spend playing outdoors, particularly unsupervised activity in and around their local community environments, is critical to their healthy development, but it is increasingly being squeezed out of children’s schedules or hindered by neighborhood barriers. The community-built environment can impact how parents’ feel about letting their children outside to play or roam freely; the quality and safety of local play amenities can also affect children’s motivation for community-based play. In order to foster increased time and freedom for children to play outdoors in their communities we need to better understand contemporary children’s outdoor play habits, and the social, cultural and environmental conditions which may be hindering neighborhood play. This study aims to explore time spent outdoors by a sample of children aged 10 to 13 years, and associations with a range of commonly reported facilitators and barriers to outdoor play. Understanding the social and environmental barriers to neighborhood play, particularly those which may be amenable to change, is key to directing effective community planning interventions and policy shifts.

## 2. Materials and Methods

### 2.1. Study Design and Participants

Surveys were conducted in the Fall of 2017 with a convenience sample of students in Grades 6 to 8 (aged 10–13 years), and their parents, across 12 elementary schools in a large county in the London, Ontario region in Canada, to examine children’s outdoor play and community mobility behaviors and perceptions. Child and parent surveys were designed to capture children’s typical outdoor and community-based activity, as well as data on a range of factors that have been highlighted in the literature to date as potential barriers or facilitators [See Supplementary Materials]. In addition to demographic and home setting characteristics, the survey captured details on children’s access to and use of home and community recreational spaces, their mode of travel to school, the degree to which they are allowed to access local destinations without an adult, time spent in structured activities such as sports or lessons, and time spent on screens or devices. Where relevant, parents were asked the same or similar questions as their children regarding outdoor activity habits and mobility license, as well as their own perceptions of community amenities and safety. The present study focuses on the time children spent outdoors in fair weather seasons (Spring, Summer and Fall), and the prominent barriers and facilitators of outdoor and community play.

Schools were categorized as urban, suburban or rural based on an examination of the demographic and built environment characteristics, such as population density, road density and housing types and density, of the school catchment area. Of the participating schools, four were designated as based in urban neighborhoods, and four each were designated suburban and rural. All students in Grades 6 to 8 at each school were eligible to participate. A total of 2240 survey consent packages were distributed to student households, each containing an optional parent version of the survey to be completed and returned. All children who completed the survey had parent/guardian consent and assented themselves to participate. Study protocols were approved by the ethics review committees of the participating school boards as well as the Centre for Addiction and Mental Health; subsequently protocols were also approved by Cornell University’s Institutional Review Board.

### 2.2. Measures

Parent survey responses were matched to their child’s responses, establishing child-parent dyads which allowed us to incorporate relevant data from both. However, child responses were used for all variables with the exception of select household and parent demographics, and for those which solicited parental perceptions or attitudes. Parent-reported variables are noted in all data tables.

#### 2.2.1. Dependent Variable

Child-reported average time spent playing outdoors per week, without adult supervision, was taken as the dependent variable. Similarly to other studies [28,34,106] average time playing outdoors per week was calculated by multiplying the typical number of days spent playing outside (from 0 to 7 days) by the typical time spent playing out on those days (less than 30 min, 30 min to less than 1 h, 1 h, 2 h, or 3 or more hours). For time spent outdoors per day, ‘less than 30 min’ was transformed as 15 min, ’30 min to less than 1 hour’ as 45 min, as ‘3 or more hours’ as 3 h.

#### 2.2.2. Independent Variables: Factors That May Impact Time in Outdoor Play

Survey variables were clustered into five exploratory models reflecting those issues thought to most commonly impact children’s outdoor play activity; each model set is outlined below. For this analysis, all questions which provided 5-point response options from Strongly Agree to Strongly Disagree were collapsed into three categories (Strongly Agree/Agree, Neither Agree nor Disagree, and Strongly Disagree/Disagree) to keep categories homogenous and to avoid categories with small sample sizes (See Results tables for affected variables). For continuous variables, unstandardized regression coefficients are reported.

##### Demographic Characteristics (Model 1)

Data was collected from children and parents on both child and household characteristics. Child surveys provided the data for child grade, child gender, ethnicity, child immigrant status, parent immigrant status and number of children in the household. Parent surveys provided the data for household income and parent educational attainment.

##### Time Available for Outdoor Free Play (Model 2)

The second model included variables related to the amount of free time children may have available for outdoor, community-based play, examining the time children spend per week in organized out-of-school physical activities (such as sports teams or dance or swimming lessons), time spent indoors on screens/devices per day, and children’s perceptions that other activities, such as sports, clubs or chores, reduce the time available for outdoor play.

##### Children’s License for Community Play and Mobility (Model 3)

The third model considered the degree of freedom children have to play independently outdoors and around their community, including whether they are allowed to travel beyond or play far from home without an adult, the potential role of child possession of a cell phone, and child perceptions of whether their parents encourage them to play outdoors. Parental attitudes towards necessary levels of child supervision were assessed using the Supervision Attributes and Risk-Taking Questionnaire (Belief in the Value of Supervision subscale) [107].

##### Socio-environmental Supports for Outdoor Play (Model 4)

The fourth model sought to test the influence of socio-environmental supports for outdoor community play on time spent outdoors, including whether children had outdoor space available at home, whether they felt there were likely to be other children nearby to play with, and whether they felt safe crossing local streets. Potential barriers such as traffic or safety concerns were also examined. Children’s community safety concerns were evaluated using the Neighborhood Safety and Crime Safety sections from the Neighborhood Environment Walkability Scale—Youth (NEWS-Y) [108]. Parent perceptions of the safety and suitability of their local community for children’s play were captured with the Neighborhood Safety and Social Cohesion subscales of the Canadian National Longitudinal Survey of Children and Youth (NLSCY) [109].

##### Child and Parent Attitudes towards Outdoor Play (Model 5)

The fifth model included questions related to child and parent attitudes towards outdoor play. Benefits and fears children’s perceive relative to outdoor play was assessed using the Attitudes Towards Outdoor Play (ATOP) scale [110]. Parent views were collected using an adapted version of the Parental Attitude Towards Their Child’s Outdoor Recreation (PACOR) tool [111].

### 2.3. Statistical Analyses

Descriptive analyses were conducted to calculated unadjusted means for all demographic and other independent variables.

All analyses used time played outdoors, in hours per week, as the dependent variable. First, bivariate multilevel regressions were run for each independent variable, including key demographic variables, to estimate the unadjusted association with time played outdoors while adjusting for the school clustering effect. Second, in the absence of an accepted, comprehensive model of factors influencing children’s outdoor play and community mobility that would support a single, hierarchical model, multiple regression models were run separately on each of the five variable sets to test associations with outdoor play, and to explore which sub-variables in each set were most predictive of time spent playing outdoors. Correlations between variables in each model set (some of which are scores from validated scales) were examined and found to be low (see Supplementary Materials for correlation tables). All variables were therefore considered to represent different theoretical concepts and were included within models to explore potentially important differences between attributes; multicollinearity within models was checked by considering the Variance Inflation Factor and the standard error of coefficients.

Each model controlled for all demographic variables outlined in Table 1. All regression analyses were conducted using Multilevel Models, with random intercept at the school level to control for the clustering effect caused by the schools. In order to interpret model results, we present model-adjusted marginal means for categorical variables and unstandardized regression coefficients for continuous variables. *p*-values for explained variance tests for each items are presented and declared significant if lower than 0.05. When more than ten values were missing for any categorical variable, we defined ‘missing’ as a new level to avoid dropping those subjects from analysis; this left 20 subjects with missing values in the dependent variable (2.4%). When fewer than ten values were missing, these were dropped from the analysis. Model diagnostic analyses were conducted where residuals were assessed for normality and homogeneity of variance; outliers and influential diagnostic measures were also considered.

## 3. Results

### 3.1. Participants

A total of 1140 children were given parental permission to participate in the study, with 1117 children assenting to participate (49.8% of eligible children). This response rate is comparable to those of other school-based youth surveys using active consent procedures, including the largest school-based survey conducted in Ontario [112,113]. The youth version of the survey was eventually completed by 1063 students (47.5%). A total of 921 parent surveys were completed and returned (41.1% of all distributed; representing 82.5% of the parents of child participants).

As this analysis aimed to use both child and their parent/guardian responses to consider influences on time children spend outdoors per week, only child surveys with a matched parent survey were used; the final sample of matched child-parent surveys was 826 (77.7% of all child participants; 36.9% of all eligible children) (See Table 1). The demographic characteristics from child surveys not included did not significantly differ from those in the final sample.

The profile of the child-parent dyads in the final sample are outlined in Table 1. The sample was 51% male, with slightly more Gr. 6 children (38%) than Gr. 7 (33%) or Gr. 8 (28%) students. Slightly more participants were from rural schools (43%) than urban (31%) or suburban (26%). Over a quarter (28%) of students identified with at least one ethnicity other than or in addition to ‘white/Caucasian’. Of child participants 18% were not born in Canada, and 24% of parent participants were themselves immigrants.

Across the child participants, the mean time spent outdoors was 7.72 h per week (See Table 2). About one third of the child participants (34.3%) typically spend 8 or more hours playing outdoors per week in fair weather seasons, and 14.8% reported spending 14 or more hours outdoors. Most children (61.3%) indicated they spend less than 8 h outside per week, and 2.1% indicated that they do not play outdoors at all. To compare, more than two-thirds (65.5%) of children reported spending 2 or more of their out-of-school leisure hours per day playing indoors on screens or digital devices, while 31.8% spend 4 or more hours per day on screens (See Table 2).

### 3.2. Demographic Characteristics (Model 1)

Table 3 presents unadjusted bivariate associations between each individual variable, including participant and household characteristics, with mean time spent outdoors per week. There was a significant bivariate association with gender (*p* < 0.001), with boys reporting an average of 8.6 h (95% CI = 8.0 to 9.2 h/week) playing outdoors per week compared to an average of 6.8 h (95% CI = 6.3 to 7.4 h/week) among girls. The significant difference remained in the regression model adjusting for other demographics.

The younger Grade 6 students spent more time playing outdoors on average (8.4 h/week; 95% CI = 7.7–9.0) than children in Grade 7 (mean = 7.5 h/week; 95% CI = 6.7–8.2), both of which spent more time out than Grade 8 students (mean = 7.1 h/week; 95% CI = 6.4–7.9). 

Significant bivariate differences also emerged by school neighborhood type in the unadjusted model (*p* = 0.006); children from rural areas spent significantly more time outdoors (8.4 h/week on average) than children from suburban communities (7.9 h/week), each of whom spent more time than children from urban neighborhoods (6.8 h/week).

The ethnic/racial background of the child was also revealed as significant (*p* = 0.018) in the unadjusted model; children identifying with at least one ethnicity other than or in addition to ‘white/caucasian’ recorded a much lower average per week (6.7 h) than children identifying only as white (8.1 h/week). Whether the child was born in or immigrated to Canada was also highly significant in the unadjusted model (*p* = 0.002); children born in Canada spent an average of 8.0 h (95% CI = 7.5–8.5 h/week) playing outdoors per week, while newcomer children spent less, an average of only 6.2 h (95% CI = 5.2–7.2 h/week) per week. These differences, however, did not remain significant in the model after adjusting for all demographics. There were no statistically significant differences in mean time outdoors based on household income, parent educational attainment, parent immigrant status, or the number of children in the household. (See Table 3).

### 3.3. Time Available for Outdoor Free Play (Model 2)

In the adjusted Model 2, controlling for all demographic variables, only differences for two variables remained significant (See Table 4). While child-reported daily screen time was inversely associated with time spent playing outdoors in the unadjusted model (*p* = 0.002), it did not remain significant in the adjusted model. However, the adjusted model highlighted that children who agreed that they were “too busy playing video games, watching TV, or on the internet, social media or texting to play outside” were much less likely to spend time outdoors (*p* < 0.001), averaging only 4.6 h/week outdoors (95% CI = 3.7–5.5 h/week) compared to the 9.5 h/week average by those who disagreed that screen time made them too busy to play out (95% CI = 8.9–10.0 h/week). Interestingly, children who reported that they typically spend 4 or more days a week playing organized physical activities such as such as swimming, hockey, karate, or gymnastics were also more likely to play outdoors, reporting a significantly higher average time outdoors (9.4 h/week; 95% CI = 8.6–10.2 h/week; *p* < 0.001) than any group reporting less time per week in organized physical activities, which ranged from only 7.5 to 6.0 h per week.

### 3.4. Children’s License for Community Play and Mobility (Model 3)

Of the variables assessing the degree of freedom or encouragement children have for unsupervised outdoor play in their communities, only two variables were predictive in the third (adjusted) model (See Table 5). Most children (85%) indicated that they are allowed to travel beyond their home property without an adult, but these children reported much higher mean times outdoors (7.9 h/week; 95% CI = 7.5–8.4 h/week; *p* < 0.001) than the 15% of children who do not have this freedom (6.0 h/week; 95% CI = 4.9–7.1 h/week). Freedom to play at further distances from home was also predictive of average time outdoors (*p* < 0.001); children who indicated they were not allowed to play far from home spent only an average of 6.3 h outdoors per week (95% CI = 5.4–7.3 h/week) compared to the 8.6 h/week averaged by those who have permission to play further from home without adult supervision (95% CI = 8.1–9.2 h/week).

### 3.5. Socio-Environmental Supports for Outdoor Play (Model 4)

The fourth adjusted model, examining variables related to perceptions of the neighborhood as safe and suitable for children’s outdoor play, highlighted three variables which remained significantly associated with outdoor play time (See Table 6). Children who agreed there were a lot of other kids in the community with whom to play spent substantially more time outdoors on average (8.6 h/week; 95% CI = 8.0–9.2 h/week; *p* = 0.005) than those who felt there were not many children nearby to play with (mean = 7.1 h/week; 95% CI = 6.4–7.8 h/week). No variables related to child or parent perceptions of neighborhood traffic and crime were predictive in the adjusted model. However, the higher parents rated their neighborhood on the Social Cohesion subscale of the Canadian NLSCY, the higher the reported time outdoors per week (beta = 0.13; 95% CI = 0.02–0.25; *p* = 0.027). Parents’ perception of whether there are good parks, playgrounds or other play spaces in their neighborhood also showed significant differences between responses, however children of parents who both agreed and disagreed with the statement spent similar levels of time outdoors (7.9 h/week; 95% CI = 7.5–8.4 and 8.3 h/week; 95% CI = 5.2–12.1), respectively; the difference related rather to children whose parents were unsure about suitable local play spaces, who spent much less time outdoors (5.8 h/week; 95% CI = 4.3–7.2 h/week) than either of the former groups. The wide confidence intervals however suggests that further investigation is required to interpret these differences, and this association may be related to another factor underscoring outdoor play time.

### 3.6. Child and Parent Attitudes towards Outdoor Play (Model 5)

The fifth and final model examined whether either child or parent attitudes towards outdoor play was associated with time children spend outdoors (See Table 7). There were significant associations with all variables in the unadjusted model, however four variables remained significant in the adjusted model. Children who agreed that they “don’t like to play outside” spent on average over 5 more hours per week outdoors (mean = 3.1; 95% CI = 1.7–4.6 h/week; *p* = 0.046) than those who disagreed with this statement (mean = 8.6; 95% CI = 8.1–9.2). Perceptions that there “are better things to do inside” also predictive lower average time outdoors (*p* < 0.001); children who agreed they preferred to play inside averaged only 3.9 h/week (95% CI = 2.9–4.8 h/week) compared to the average 9.8 h/week (95% CI = 9.2–10.4) spent outdoors by children who disagreed with the premise.

Children who perceived more benefits of outdoor play were more likely to spend outdoors per week (beta = 1.18; 95% CI = 0.44–1.92; *p* = 0.002), and children who reported higher levels of fears or concerns related to outdoor play spent significantly less time outdoors on average (beta= −0.96; 95% CI= −1.54–−0.39); *p* = 0.001).

## 4. Discussion

This analysis sought to capture outdoor activity behaviors and perceptions of a diverse range of Canadian children, and to examine several of the major factors thought to influence contemporary children’s time and freedom to play outdoors. Community-level barriers amenable to planning policy or practice changes are of particular interest.

Strong predictors of time outdoors within each of the models tested confirm that the factors influencing children’s outdoor and neighborhood activity include diverse objective measures as well as subjective perceptions of the social, cultural and physical environment, and reinforce the need for a socio-ecological approach to examining children’s activity and mobility behaviors.

### 4.1. The Influential Role of Child Age and Gender

Analyses revealed that the mean self-reported time spent outdoors by child participants represents the equivalent of about 1.1 h of outdoor play per day. This average is substantially lower than several studies with comparable age groups which reported closer to 1.5 to 2 h outdoors per day on average [28,64,106] though more than the 42.4 min per day reported for early adolescent (13–14 years) samples only [64]. In contrast with a study of weekday outdoor play time with a comparable population of Canadian children [33], a higher proportion of children in this study (63.3% vs. 55.1%) spent the equivalent of 1 h or less outdoors per day on average, but also reported a higher proportion of children (14.8% vs. 7.7%) spending an average of 2 or more hours outdoors per day.

While many demographic variables showed significant bivariate associations with time spent outdoors, the multiple regression models highlighted that child age and gender remain the most predictive. These results echo findings from the literature to date; child age has been consistently associated with outdoor play time, with time outdoors declining steadily as children transition from middle childhood to adolescence. Despite the typically concurrent increase in independent community mobility as children mature, some scholars have explained the decline in time spent outdoors as children age a result of increased school and homework loads [114] or a shift in interests towards more social or challenging activities, for which community outdoor spaces may not appeal or feel suitable for older children or teens [36,67,72,77]. The differences in participants’ outdoor play time by age even within this fairly narrow age range is an important reminder that not only do children’s play and recreation interests shift significantly across childhood and adolescence, but that the neighborhood conditions and perceptions which are influencing young people’s activity also shift. Community provisions for outdoor play tend to focus primarily on the interests of younger children, often in the form of play structures, reducing the appeal of neighborhood spaces as children age [72,115]. Child-friendly planning strategies need to do more to address the diversity of play interests across the age spectrum.

Gender differences in time spent outdoors have also been strongly and fairly consistently reported across Western countries, illustrating that despite cultural shifts towards viewing the capacities and activity needs of all children more equitably regardless of gender, boys in the 7–13-year-old range still generally spend more time playing outdoors and in community places beyond their home than girls. This enduring gender difference in time spent outdoors, often theorized as being linked to higher restrictions on girls’ independent activity and movement due to safety concerns or sociocultural norms bears more research attention. Child-friendly community planning strategies may be particularly effective at reducing this gender gap; planning practices and policies that focus on increasing child and parent perceptions of neighborhood safety and improving child-friendly infrastructure such as safe sidewalks and pathways to local destinations, may facilitate more outdoor activity in the community by girls. Persistent gender differences in outdoor play and mobility also suggests more qualitative work should direct attention to understanding community amenities and conditions that may particularly appeal to the needs and interests of girls and the concerns of their parents. Future work should also explore whether gender differences in time and freedom for outdoor play intersect with newcomer status or ethnic/cultural background; girls from newcomer families or from ethnic minorities may face more restrictions on their neighborhood activity or barriers to safe, independent community play [64,116]. Several studies have also established that children and adults from different cultural backgrounds demonstrate varying preferences and motivations for outdoor recreation [117,118] which should influence community planning practices; however there is still little work investigating inter- or intra-ethnic differences in children’s outdoor play, including opportunities and preferences. Growing population diversity in Western countries requires contemporary child-friendly planning practices to address differences in use and preference in order to provide culturally appropriate outdoor spaces and opportunities for all children and families.

### 4.2. Neighborhood Type and Other Child and Household Demographics

While neighborhood setting did not emerge as predictive in the adjusted demographics model, the significant bivariate association (unadjusted) with time outdoors merits further exploration as it may have substantial implications for child-friendly community planning. Evidence from other studies suggest the degree of urbanization impacts children’s community-based independent mobility and active transportation [59,119,120,121], both of which have been tied themselves to levels of outdoor neighborhood play, especially activity without adult supervision. However, examining differences in outdoor play in relation to neighborhood type or degree of urbanization has only recently gained attention in the research, and so evidence to date is limited. It may be that children from urban and rural communities face different types of barriers to outdoor play that are specific to their differing social and physical environmental conditions; these differential impacts on community outdoor play need to be better unpacked, including how they may intersect with child age, gender or IM permissions, to understand how child-friendly community planning policies need to be tailored to address barriers inherent to different neighborhood types.

Despite associations reported in previous studies between outdoor play time and various socioeconomic variables, this analysis found no statistical association between time outdoors and household income or parent education attainment, or the number of children in the household. The lack of association with SES-related variables in this analysis may be related to limited variability in the participant sample, which may have oversampled higher SES households.

### 4.3. Time Available for Outdoor Play

It has been hypothesized that increasing levels of engagement on screens or digital devices may be in part responsible for declining levels of outdoor and community-based play across recent decades [6,28]. The Canadian Society for Exercise Physiology (CSEP) 24-Hour Movement Guidelines recommend that young people aged 5 to 17 years should spend no more than 2 hours per day of sedentary behavior, particularly recreational screen time [122]. Over two-thirds of children in this study spent more than the recommended time in sedentary, screen-based activity per day and almost a third spend 4 or more hours per day on screens. There were significant bivariate associations with the amount of time children spent on screens or digital devices per day; the mean time spent outdoors per week was highest for children who reported only 30 min of screen time per day, and then consistently declined as children spent more time per day indoors on screens. Time reported indoors on screens did not remain predictive in the adjusted model yet a child’s perception of whether screen-based activities compromised their time for outdoor play was predictive; children who agreed they were too busy with screen-based activities for outdoor spent significantly less time outdoors on average. These results may suggest that screen-based activities are more appealing than outdoor play to some children, and they are choosing these digital activities over outdoor play; other children are perhaps choosing more of a balance of screen and outdoor play time. Overall, screen time does appear to be related to outdoor play time, but it may be affecting some children’s time outdoors more than others depending on their preferences or their level of IM. Earlier studies by Loebach and Gilliland [72,75] suggest that greater time indoors and on screens may be related to a lack of appealing nearby play opportunities or community destinations. Additional qualitative work on children’s attitudes and preferences related to both screen-based and outdoor activities would help to better illuminate the nuances of these connections. Understanding neighborhood amenities and environments which appeal to children’s interests can help to direct more child-friendly planning efforts and may work to offset rising levels of indoor screen time.

The positive association with free time spent outdoors and time spent in organized physical activities was contrary to initial hypotheses, particularly as the latter would reduce the amount of free time per week children have for unstructured outdoor activity. For some children, they may have reported the time outdoors in the organized activity as part of their weekly outdoor play time. However, a case can also be made that children who spend more time engaged in organized physical activities are more interested in and motivated to spend their leisure time playing outdoors, particularly in active pursuits. Leisure time is an opportunity for children to choose the activities in which they engage, and this choice is affected by preference for and satisfaction with physical versus sedentary activity [6,123,124]. Children who participate in and enjoy organized physical activities may therefore be more motivated and equipped to choose and engage in physical activities in their ‘off hours’ [125], which are well supported in outdoor environments [126,127,128]. Children who are regularly engaged in sports have been found to be more likely to play outdoors during the after-school period [98]. In addition, children who enjoy participating in organized physical activities, such as team sports, may have friends who are also more inclined to be active outdoors in their free time; peers with similar activity preferences, including during outdoor play, have been shown to positively influence outdoor play and physical activity [129,130].

### 4.4. Permission for Unsupervised Outdoor and Community Activity

The degree of freedom children have to play outdoors in the community without adult supervision (IM) has been highly correlated in other studies with parents’ perceptions of how safe the neighborhood is for children, particularly from traffic, strangers and local crime. While variables associated with parent perceptions of neighborhood safety were not predictive of children’s time outdoors in this study, child-reported parental restrictions which limited children’s access to community outdoor spaces beyond their home property, whatever the motivation for these restrictions, was predictive of lower levels of outdoor play. Evidence that many of the 10–13-year-old participants still experience significant barriers to unsupervised play corroborates trends in Western countries that see children gaining independent mobility at much older ages than in previous generations as well as the link between greater IM and higher levels of outdoor play. A qualitative study by Brockman et al. [67] revealed that a primary motivator for outdoor play for some children was the opportunity to spend some time outside of adult control and supervised activities. Parents are largely the gatekeepers in terms of children’s IM and need to feel more comfortable with the neighborhood as a safe space of their children. Both public health messaging and community planning practices can work to reinforce the benefits of independent, outdoor play and address neighborhood conditions that may be causing parents concern.

### 4.5. Neighborhood Social and Environmental Conditions

One neighbourhood-level factor that may make communities feel safer to parents and more appealing to children is the presence of other children. This study’s positive association between time outdoors with children’s perception of other kids nearby to play with confirms findings from other research to date. A number of studies have shown that one of the key motivators for outdoor play among children was the chance to spend time with friends [67,131,132]. Loebach and Gilliland [72] found that most 9 to 12 year old participants’ independent neighborhood activity was carried out in the company of other children, and community parks and playgrounds often served as the common meeting place with other young people. Children have also been found to spend more and longer times playing outdoors if they are with some friends [98,115,133,134]. A meta-analysis of qualitative outdoor play studies by Lee et al. [36] also suggests that when there are fewer other young people in the nearby community for their children to play with, parents were more reluctant to let their children play outdoors in the neighborhood, feeling that there was some increased level of safety with numbers. Children playing in the neighborhood seems to beget children playing in the neighborhood; parents are often reassured when their child is not alone, and children are able to engage in the social recreational time they crave. While planning practices cannot ensure there will be children nearby to play with, attention to child-friendly infrastructure such as safe and plentiful pedestrian pathways to community public spaces, paired with diverse amenities and youth-focused community programs, can provide children with more, and more accessible, opportunities to connect on their own with neighborhood peers.

This study also highlighted the role of parents’ perceptions of neighborhood conditions for play. Parent attitudes towards outdoor play, as well as neighbourhood safety and suitability for play have been found in other studies be significant predictors of the time and freedom children have to spend recreating outdoors yet the current study found that parent perceptions of neighborhood safety were not significantly associated with outdoor play time. However, children were more likely to play outdoors if their parents perceived a high level of social cohesion within their community; social cohesion can be viewed as a proxy of sorts for perceptions of neighborhood social safety, as parents who feel that they can trust their neighbors to look out for and be a safe haven for their children in cases of trouble may be more inclined to allow their children to play out in the community. Other studies have shown that parents’ neighborhood safety concerns are evident in the degree of adult supervision they impose on children’s outdoor activity, or the requirement to carry a cell phone [36], however, neither parental attitude towards supervision nor child possession of a cell phone was significantly associated with time outdoors in this study. We can take away, however, the importance of a sense of community and social cohesion in facilitating outdoor play; community-level policies and programs which aim to facilitate these bonds could support greater neighborhood play. Engaging both parents and children in community planning conversations may help to highlight mechanisms for increasing a local sense of community.

### 4.6. Children’s Perceptions of Outdoor Play

Finally, regression analyses reinforced that children’s own attitudes towards outdoor play are significant predictors of time spent out. Children who agreed that playing outside makes them healthier, helps them learn new things, gives them the opportunity to explore, and to regulate their feelings, were more likely to report spending time playing outdoors. Conversely, the more children reported different fears associated with playing outdoors, such as being afraid of strangers, wild animals and insects, or getting hurt, the less time they reported outdoors. However, parent attitudes towards outdoor play were not predictive in the final model, reminding us that while parents need to provide children with permission to play outdoors, the child’s own interest in playing outdoors may be a greater driver of actual behavior. Safe, accessible and high-quality play spaces may be made available in a community, but they will not be utilized by children if they are not motivated by the benefits of outdoor play or able to overcome any fears related to playing outdoors. Here, we see where household and public health messaging espousing the benefits and safety of outdoor play has a role to play in promoting community-based activities. Beyer et al. [135] suggest that environmental education programs and greater familiarity with neighborhood environments may be one way to help children overcome any aversions and promote awareness of community play opportunities. Awareness and education programs paired with child-friendly planning practices may facilitate increased comfort with and engagement in community-based outdoor play.

### 4.7. Strengths and Limitations

An asset of this study was the investigation of a diverse range of social, cultural and environmental factors thought to influence time children spend playing outdoors. Predictive factors emerged from regression models with each variable set, confirming that the issues impacting outdoor play behaviors are many and complex, and that both research and planning approaches must consider the diverse objective and subjective factors influencing outdoor and community-based activity. Another improvement over recent studies was the inclusion of child-reported time outdoors versus reliance on parent estimates of outdoor play which may over or underestimate actual time. However, the use of a time value calculated from interval data may also compromise the accuracy of the outdoor play time variable. The study also benefitted from the use of child-parent dyads; while child responses provided the majority of study variables, the ability to link parent responses to a specific child participant allowed us to connect more socioeconomic characteristics such as household income, but also to consider the role of parent perceptions and attitudes on the behaviors and perceptions of each child. While a range of parent perceptions were included in the analysis, we acknowledge that there may be other parent beliefs or practices influencing play which were not included in this study.

The study is in part limited due to its cross-sectional nature, and the use of a convenience sample of schools from a single county in Ontario, Canada. While many variables were included for consideration in the analyses, the participant sample was not large enough to test a single, hierarchical model which may be able to highlight which factors are the strongest predictors of outdoor play. However, individually modelling factors thought to influence outdoor play highlighted multiple predictors which in turn provide multiple potential points of focus for directing planning interventions and policies to mitigate barriers and amplify facilitators of outdoor and neighborhood play.

Future work will attempt to dig deeper into a number of the predictive factors which emerged from this analysis, particularly gender, to better understand how child and parent perceptions, as well as community social and environmental conditions may be differentially impacting or moderating outdoor play across different child populations. These analyses will also integrate objective measures of the neighborhood-built environment to confirm or extend findings from this paper around child and parent neighborhood perceptions.

## 5. Conclusions

Free play outdoors at home and around their communities has been linked to significant health and developmental benefits for children, including increased physical activity, environmental and social competence, creativity, and spatial skills. These benefits are generally reinforced when this activity takes place independently, without the direct supervision of adults. The dramatic drop in Western children’s time and freedom to play outdoors and travel independently around their neighborhoods not only limits children’s opportunity to capitalize on these benefits but potentially compromises the instilling of key skills and competencies. This analysis of barriers and facilitators impacting the outdoor and community-based free play of a sample of 9- to 12-year-old Canadian children highlights a number potential points of focus for child-friendly community planning efforts. Analyses revealed that most of the participant children are spending less time outdoors and more time on screens that child health guidelines recommend. The association between interest in screen-based activities and lower time outdoors reminds us that outdoor play activities may not hold children’s interest as much as digital play particularly when independent mobility and peer interaction is limited and the nearby outdoor environment provides few appealing or accessible play opportunities. Associations also confirm the influential role of both child and parent perceptions of social and environmental conditions of the nearby community, and attitudes towards outdoor play, which can be addressed in part through informed planning practices and public messaging.

This study lends additional weight to the existing scholarship, confirming that multiple socio-environmental barriers, including lack of access to appealing outdoor play environments and conditions at home and their neighborhood, are interacting to hinder some children’s outdoor and community play, and underscoring the importance of multi-pronged planning strategies to provide the conditions and infrastructure necessary to create local environments which can support outdoor play. Professional planners and municipal policymakers can utilize such analyses in combination with engagement with both children and parents to understand and then target the play needs and barriers specific to local neighborhoods, and to diverse community populations. These community-level planning practices, especially when paired with outdoor play awareness campaigns and programs, can be effectively marshalled to create communities which are genuinely child- and play-friendly.

## Figures and Tables

**Table 1:** Participant Characteristics.

Participants		*n*	%
Child-parent dyads		826	
Gender	Girls	404	48.9%
	Boys	422	51.1%
Grade	Gr. 6	317	38.4%
	Gr. 7	275	33.3%
	Gr. 8	234	28.3%
Neighborhood Type	Urban	258	31.2%
	Suburban	212	25.7%
	Rural	356	43.1%
Household Income per Year	Less than USD 60,000	169	20.5%
	USD 60,000 to 100,000	164	19.9%
	More than USD 100,000	284	34.4%
	Prefer not to answer	145	17.6%
	Missing	64	7.7%
Parent Education	High school or less	107	13.0%
	College diploma or some university	336	40.7%
	University degree	366	44.3%
	Missing	17	2.1%
Child Immigrant Status	Born in Canada	675	81.7%
	Immigrant to Canada	147	17.8%
Parent Immigrant Status	Born in Canada	617	74.7%
	Immigrant to Canada	195	23.6%
	Missing	14	1.7%
Child Ethnicity	Identified as White/Caucasian	567	68.6%
	Identified as a least one Non-White/Caucasian ethnicity	230	27.8%
	Missing	29	3.5%
No. of Children in Household	1	136	16.5%
	2	415	50.2%
	3	171	20.7%
	4 or more	76	9.2%
	Missing	28	3.4%

**Table 2:** Participant Children’s Time Outdoors and On Screens.

	Outdoor Play (Hours per Week) *n* = 826			Indoor Screen Time (Hours per Day) *n* = 826	
Average	7.72				
	*n*	%		*n*	%
I don’t play outdoors	17	2.1	No time	14	1.7
1 h or less	55	6.7	30 min	87	10.5
>1 h to <4 h	249	30.1	1 h	158	19.1
>4 h to <8 h	202	24.5	2 to 3 h	278	33.7
>8 h to <14 h	161	19.5	4 to 6 h	175	21.2
More than 14 h	122	14.8	7 or more h	88	10.7
Missing	20	2.4	Missing	26	3.1
Proportion meeting > 1 h per day outdoors recommendation		~34.3%	Proportion exceeding < 2h per day recommendation		65.5%

**Table 3:** Demographic Characteristics: Descriptives and Model 1 Results ^1^.

Demographics		Unadjusted Model ^+^				Adjusted Model ^++^		
		*n* (%)	Mean	95% CI	*p*-Value	Mean	95% CI	*p*-Value
Grade	6	317 (38)	8.4	7.6–9.1	0.026 *	8.4	7.7–9.0	0.046 *
	7	275 (33)	7.3	6.5–8.2		7.5	6.7–8.2	
	8	234 (28)	7.0	6.2–7.9		7.1	6.4–7.9	
Gender	Boys	422 (51)	8.5	7.8–9.2	<0.001 ***	8.6	8.0–9.2	<0.001 ***
	Girls	404 (49)	6.7	6.0–7.4		6.8	6.3–7.4	
Income per Year	< USD 60,000	169 (20)	7.5	6.3–8.7	0.234	7.1	5.9–8.3	0.376
	USD 60–99,999	164 (20)	8.2	7.4–8.9		8.2	7.5–8.8	
	USD 100,000+	284 (34)	7.2	6.5–7.9		7.5	6.8–8.2	
	Prefer not to answer	145 (18)	8.4	5.4–11.5		7.4	3.5–11.3	
	Missing	64 (8)	7.3	6.3–8.3		7.4	6.5–8.4	
Education	High school or less	107 (13)	7.0	6.0–8.1	0.350	7.1	6.1–8.0	0.391
	College/Some Univ.	336 (41)	8.0	7.2–8.9		8.0	7.3–8.8	
	Graduated university	366 (44)	7.7	6.7–8.8		7.8	6.8–8.7	
	Missing	17 (2)	8.5	6.9–10.1		8.7	7.0–10.3	
Neighbor-hood Type	Urban	258 (31)	6.8	6.0–7.5	0.006 **	7.0	6.2–7.9	0.171
	Suburban	212 (26)	7.8	7.0–8.7		7.9	7.1–8.8	
	Rural	356 (43)	8.4	7.7–9.0		8.1	7.4–8.8	
Ethnicity	White	567 (69)	8.1	7.5–8.7	0.018 *	8.0	7.4–8.5	0.389
	Non-White	230 (28)	6.7	5.9–7.6		7.2	6.2–8.2	
	Missing	29 (4)	7.1	4.8–9.3		7.2	4.9–9.4	
Child Immigrant Status	Born in Canada	675 (82)	8.0	7.5–8.5	0.002 **	7.9	7.4–8.4	0.127
	Immigrant to Canada	147 (18)	6.2	5.2–7.2		6.8	5.6–8.1	
Parent Immigrant Status	Born in Canada	617 (75)	7.9	7.4–8.5	0.069	7.7	7.1–8.2	0.843
	Immigrant to Canada	195 (24)	6.8	5.9–7.7		7.8	6.7–8.9	
	Missing	14 (2)	9.0	5.6–12.3		8.8	5.1–12.5	
Number of children in the household	1	136 (16)	7.7	6.6–8.8	0.499	7.9	6.9–8.9	0.242
	2	415 (50)	7.4	6.7–8.1		7.5	6.9–8.1	
	3	171 (21)	7.5	6.5–8.5		7.5	6.6–8.4	
	4 to 8	76 (9)	8.7	7.3–10.2		9.2	7.9–10.6	
	Missing	28 (3)	8.2	5.9–10.6		7.9	5.1–10.6	

^1^ Means are in hours per week. * *p* < 0.05 ** *p* < 0.01 *** *p* < 0.001. ^+^ Raw sample size and mean time outdoors adjusted for school clustering only. *p*-values are for test of mean differences across levels of the demographics (similar to ANOVA). *p*-values are calculated from Wald Chi-square statistics from multilevel models where an intercept is fitted at the school level. Demographics are tested separately. ^++^ Means are estimated marginal means from a multilevel model that includes school level intercept and all demographics as independent variables simultaneously. *p*-values are calculated from Wald Chi-square statistics from the model.

**Table 4:** Time Available for Outdoor Free Play: Descriptives and Model 2 Results. Adjusted model controls for demographic variables in Model 1 (Table 3) ^1^.

		Unadjusted Model ^+^				Adjusted Model ^++^		
		*n* (%)	Mean	95% CI	*p*-Value	Mean	95% CI	*p*-Value
Too busy with sports	Strongly Disagree/Disagree	454 (56)	7.7	7.0–8.4	0.902	8.0	7.4–8.5	0.35
	Neither Agree or Disagree	217 (27)	7.5	6.6–8.4		7.2	6.5–8.0	
	Strongly Agree/Agree	147 (18)	7.6	6.5–8.7		7.5	6.5–8.5	
Too busy with clubs	Strongly Disagree/Disagree	677 (82)	8.1	7.5–8.6	<0.001 ***	7.9	7.4–8.3	0.312
	Neither Agree or Disagree	92 (11)	5.8	4.5–7.1		7.0	5.8–8.2	
	Strongly Agree/Agree	40 (5)	5.5	3.6–7.4		6.4	4.6–8.3	
	Missing	17 (2)	6.7	3.8–9.7		7.0	3.7–10.2	
Too busy with chores	Strongly Disagree/Disagree	621 (76)	7.9	7.3–8.5	0.104	7.7	7.2–8.1	0.734
	Neither Agree or Disagree	137 (17)	7.0	5.9–8.0		7.5	6.5–8.5	
	Strongly Disagree/Disagree	621 (76)	7.9	7.3–8.5	0.104	7.7	7.2–8.1	0.734
Too busy on screens	Strongly Disagree/Disagree	411 (50)	9.6	9.0–10.3	<0.001 ***	9.5	8.9–10.0	<0.001 ***
	Neither Agree or Disagree	218 (26)	6.8	6.0–7.7		7.0	6.2–7.7	
	Strongly Agree/Agree	186 (23)	4.5	3.6–5.4		4.6	3.7–5.5	
	Missing	11 (1)	6.7	3.3–10.1		4.7	0.2–9.1	
Days per week in organized physical activities	Never	164 (20)	5.7	4.7–6.7	<0.001 ***	6.0	5.1–7.0	<0.001 ***
	< once a week	51 (6)	6.8	5.2–8.6		7.3	5.7–8.9	
	1–3 times a week	365 (44)	7.5	6.8–8.2		7.5	6.9–8.0	
	4 or more times a week	231 (28)	9.6	8.7–10.5		9.4	8.6–10.2	
	Missing	15 (2)	7.3	4.1–10.6		5.5	2.0–8.9	
Daily indoor screen/digital time	None	14 (2)	6.9	3.7–10.1	0.002 **	5.6	2.5–8.6	0.178
	1/2 h/day	87 (11)	10.0	8.7–11.4		8.6	7.4–9.9	
	1 h/day	158 (19)	8.2	7.1–9.2		7.4	6.5–8.3	
	2–3 h/day	278 (34)	7.3	6.5–8.1		7.2	6.5–7.9	
	4–6 h/day	175 (21)	7.3	6.4–8.3		8.0	7.1–8.8	
	7+ h/day	88 (11)	6.3	5.0–7.6		8.1	6.8–9.3	
	Missing	26 (3)	8.3	5.8–10.7		9.4	6.8–11.9	

^1^ Means are in hours per week. ** *p* < 0.01 *** *p* < 0.001. ^+^ Raw sample size and mean time outdoors adjusted for school clustering only. *p*-values are for test of mean differences across levels of the variables (similar to ANOVA). *p*-values are calculated from Wald Chi-square statistics from multilevel models where an intercept is fitted at the school level. Each variable in the set was tested separately. ^++^ Means are estimated marginal means from a single multilevel model that includes school level intercept and all demographics as control variables as well as all the Set 2 variables simultaneously. *p*-values are calculated from Wald Chi-square statistics from the model.

**Table 5:** Children’s License for Community Activity and Mobility: Descriptives and Model 3 Results. Adjusted model controls for demographic variables in Model 1 (Table 3) ^1^.

		Unadjusted Model ^+^				Adjusted Model ^++^		
		*n* (%)	Mean	95% CI	*p*-Value	Mean	95% CI	*p*-Value
Not Allowed to Travel Beyond Home w/o Adult	No	705 (85)	8.1	7.5–8.7	<0.001 ***	7.9	7.5–8.4	0.002 **
	Yes	121 (15)	5.2	4.1–6.4		6.0	4.9–7.1	
Not Allowed to Play Far From Home w/o Adult	Strongly Disagree/Disagree	456 (56)	8.8	8.2–9.4	<0.001 ***	8.6	8.1–9.2	<0.001 ***
	Neither Agree or Disagree	188 (23)	6.4	5.5–7.3		6.5	5.6–7.3	
	Strongly Agree/Agree	172 (21)	6.0	5.0–6.9		6.3	5.4–7.3	
Allowed to Cross Main Roads w/o adult	No	163 (20)	6.8	5.8–7.8	0.147	7.7	6.7–8.7	0.894
	Yes	647 (78)	7.9	7.3–8.5		7.7	7.2–8.2	
	Missing	16 (2)	7.9	4.5–11.2		6.9	3.6–10.2	
Child Has Cell Phone	No	323 (40)	7.4	6.6–8.2	0.387	7.5	6.8–8.2	0.511
	Yes	494 (60)	7.8	7.1–8.4		7.8	7.3–8.3	
Parents Encourage OP	Strongly Disagree/Disagree	47 (6)	7.2	5.4–9.0	0.250	7.7	6.0–9.5	0.583
	Neither Agree or Disagree	127 (15)	7.4	6.3–8.5		7.8	6.7–8.8	
	Strongly Agree/Agree	636 (77)	7.8	7.2–8.4		7.7	7.2–8.2	
	Missing	16 (2)	4.8	1.7–8.0		5.3	2.0–8.7	
Parent: Parental attitude towards level of children supervision (scale 0–100) ^		814 (100)	0.002	−0.04–0.05	0.916	0.04	−0.01–0.09	0.114

^1^ Means are in hours per week. ** *p* < 0.01 *** *p* < 0.001. ^+^ Raw sample size and mean time outdoors adjusted for school clustering only. *p*-values are for test of mean differences across levels of the variables (similar to ANOVA). *p*-values are calculated from Wald Chi-square statistics from multilevel models where an intercept is fitted at the school level. Each variable in the set was tested separately. ^++^ Means are estimated marginal means from a single multilevel model that includes school level intercept and all demographics as control variables as well as all the Set 3 variables simultaneously. *p*-values are calculated from Wald Chi-square statistics from the model. ^ Continuous variable. The mean is the model coefficient (slope).

**Table 6:** Socio-environmental Supports for Outdoor Play: Descriptives and Model 4 Results. Adjusted model controls for demographic variables in Model 1 (Table 3) ^1^.

		Unadjusted Model ^+^				Adjusted Model ^++^		
		*n* (%)	Mean	95% CI	*p*-Value	Mean	95% CI	*p*-Value
Avail. of outdoor space at home	No	53 (6)	5.0	3.4–6.7	0.001 **	6.6	4.7–8.4	0.177
	Yes	772 (94)	7.9	7.3–8.4		7.9	7.4–8.3	
Neighborhood Traffic Limits OP	Strongly Disagree/Disagree	640 (78)	8.1	7.6–8.7	<0.001 ***	8.0	7.6–8.5	0.164
	Neither Agree or Disagree	128 (16)	6.2	5.1–7.3		6.9	5.8–8.0	
	Strongly Agree/Agree	54 (7)	5.6	4.0–7.3		7.0	5.2–8.8	
Kids in the neighborhood to play with	Strongly Disagree/Disagree	299 (37)	6.8	6.0–7.6	<0.001 ***	7.1	6.4–7.8	0.005 **
	Neither Agree or Disagree	163 (20)	6.8	5.8–7.8		7.2	6.3–8.1	
	Strongly Agree/Agree	356 (44)	8.8	8.1–9.6		8.6	8.0–9.2	
Neighborhood Traffic Limits Mobility	Strongly Disagree/ Disagree	591 (72)	7.9	7.2–8.5	0.407	7.6	7.1–8.1	0.704
	Neither Agree or Disagree	143 (17)	7.3	6.3–8.4		8.1	7.0–9.1	
	Strongly Agree/Agree	78 (9)	6.9	5.5–8.3		8.4	7.0–9.8	
	Missing	14 (2)	6.3	3.0–9.6		9.6	1.5–17.8	
Feel Safe Crossing Local Streets	Strongly Disagree/Disagree	44 (5)	8.2	6.3–10.1	0.112	9.5	7.5–11.5	0.379
	Neither Agree or Disagree	93 (11)	6.4	5.1–7.7		7.6	6.3–8.9	
	Strongly Agree/Agree	670 (81)	7.8	7.2–8.4		7.7	7.3–8.2	
	Missing	19 (2)	5.9	3.2–8.7		8.0	3.6–12.4	
Parent: Neighborhood Safe for Kids During the Day	Strongly Disagree/Disagree	14 (2)	5.8	2.6–9.0	0.083	8.6	5.2–12.1	0.763
	Neither Agree or Disagree	46 (6)	6.0	4.2–7.8		7.3	5.4–9.1	
	Strongly Agree/Agree	760 (93)	7.8	7.2–8.4		7.8	7.4–8.2	
Parent: Neighborhood Has Good Places for Play	Strongly Disagree/Disagree	67 (8)	7.8	6.3–9.4	<0.001 ***	8.3	6.8–9.9	0.017 *
	Neither Agree or Disagree	70 (9)	4.8	3.3–6.2		5.8	4.3–7.2	
	Strongly Agree/Agree	682 (83)	7.9	7.3–8.5		7.9	7.5–8.4	
Child Perceived Neighbourhood Crime ^		790	−0.16	−0.26–−0.06	0.002 **	−0.11	−0.22- 0.01	0.062
Parent: Perception of Neighbourhood Cohesion ^		799	0.22	0.11–0.32	<0.001 ***	0.13	0.02- 0.25	0.027 *

^1^ Means are in hours per week. * *p* < 0.05 ** *p* < 0.01 *** *p* < 0.001. ^+^ Raw sample size and mean time outdoors adjusted for school clustering only. *p*-values are for test of mean differences across levels of the variables (similar to ANOVA). *p*-values are calculated from Wald Chi-square statistics from multilevel models where an intercept is fitted at the school level. Each variable in the set was tested separately. ^++^ Means are estimated marginal means from a single multilevel model that includes school level intercept and all demographics as control variables as well as all the Set 4 variables simultaneously. *p*-values are calculated from Wald Chi-square statistics from the model. ^ Continuous variable. The mean is the model coefficient (slope).

**Table 7:** Child and Parent Attitudes Towards Outdoor Play: Descriptives and Model 5 Results. Adjusted model controls for demographic variables in Set 1 (Table 3) ^1^.

		Unadjusted Model ^+^				Adjusted Model ^++^		
		*n* (%)	Mean	95% CI	*p*-Value	Mean	95% CI	*p*-Value
Playing outside is fun and exciting	Strongly Disagree/Disagree	38 (5)	2.9	0.9–4.8	<0.001 ***	7.0	4.8–9.2	0.672
	Neither Agree or Disagree	95 (12)	4.6	3.4–5.8		7.4	6.1–8.7	
	Strongly Agree/Agree	690 (84)	8.4	7.8–8.9		7.9	7.5–8.3	
I don’t like to play outside	Strongly Disagree/Disagree	668 (82)	8.6	8.1–9.2	<0.001 ***	8.1	7.6–8.5	0.046 *
	Neither Agree or Disagree	86 (11)	4.3	3.0–5.6		6.2	4.9–7.5	
	Strongly Agree/Agree	65 (8)	3.1	1.7–4.6		7.0	5.2–8.9	
I don’t like to play outside because it is boring	Strongly Disagree/Disagree	630 (77)	8.7	8.1–9.3	<0.001 ***	8.1	7.6–8.5	0.159
	Neither Agree or Disagree	118 (14)	5.0	3.9–6.1		7.0	5.9–8.1	
	Strongly Agree/Agree	71 (9)	3.1	1.7–4.5		6.5	4.7–8.3	
I don’t like to play outside because it is too dirty	Strongly Disagree/Disagree	720 (87)	8.0	7.4–8.5	0.001 ***	7.7	7.3–8.1	0.894
	Neither Agree or Disagree	67 (8)	5.7	4.2–7.2		8.1	6.7–9.5	
	Strongly Agree/Agree	28 (3)	4.4	2.2–6.7		8.5	6.2–10.9	
	Missing	11 (1)	8.0	4.4–11.6		8.1	3.8–12.5	
I don’t like to play outside because I will get too hot/sweaty	Strongly Disagree/Disagree	637 (78)	8.5	8.0–9.0	<0.001 ***	8.0	7.6–8.4	0.160
	Neither Agree or Disagree	126 (15)	5.4	4.3–6.5		6.8	5.7–7.9	
	Strongly Agree/Agree	55 (7)	4.0	2.4–5.6		7.5	5.8–9.3	
I don’t like to play outside because I will get too cold	Strongly Disagree/Disagree	565 (69)	8.5	7.9–9.1	<0.001 ***	7.8	7.3–8.3	0.995
	Neither Agree or Disagree	172 (21)	6.6	5.7–7.6		7.8	6.9–8.7	
	Strongly Agree/Agree	84 (10)	4.7	3.4–6.0		7.8	6.5–9.2	
I don’t like to play outside because there are better things to do inside	Strongly Disagree/Disagree	426 (52)	9.8	9.2–10.4	<0.001 ***	8.8	8.2–9.4	<0.001 ***
	Neither Agree or Disagree	153 (31)	6.4	5.6–7.1		6.7	6.0–7.4	
	Strongly Agree/Agree	241 (17)	3.9	2.9–4.8		6.7	5.4–8.0	
Parent: Parent attitudes towards playing outdoors (PACOR) ^		802	0.1	0.1	<0.001 ***	0.03	−0.02–0.07	0.205
Child Perceived Benefits of Outdoor Play (ATOP) ^		805	2.8	2.8	<0.001 ***	1.18	0.44–1.92	0.002 **
Child Perceived Fears of Outdoor Play (ATOP) ^		803	−1.8	−1.8	<0.001 ***	−0.96	−1.54–−0.39	0.001 **

^1.^ Means are in hours per week. * *p* < 0.05 ** *p* < 0.01 *** *p* < 0.001. ^+^ Raw sample size and mean time outdoors adjusted for school clustering only. *p*-values are for test of mean differences across levels of the variables (similar to ANOVA). *p*-values are calculated from Wald Chi-square statistics from multilevel models where an intercept is fitted at the school level. Each variable in the set was tested separately. ^++^ Means are estimated marginal means from a single multilevel model that includes school level intercept and all demographics as control variables as well as all the Set 5 variables simultaneously. *p*-values are calculated from Wald Chi-square statistics from the model. ^ Continuous variable. The mean is the model coefficient (slope).

## Data Availability

At the time of publication the data presented in this study are available only on request from the corresponding author. The data are not publicly available to protect the identity of child participants.

## Supplementary Materials

The following are available online at https://www.mdpi.com/article/10.3390/ijerph18073617/s1, Table S1: Spearman Correlation matrix for Model 2: Time Available for Outdoor Free Play, Table S2: Spearman Correlation Matrix for Model 3: Children’s License for Community Activity & Mobility, Table S3: Spearman Correlation Matrix for Model 4: Socio-environmental Supports for Outdoor Play, Table S4: Spearman Correlation Matrix for Model 5: Child and Parent Attitudes Towards Outdoor Play. Figures S1–S5: Graphs of Estimated Marginal Means. Tool S1: Outdoor Play Survey—Youth Version, Tool S2: Outdoor Play Survey—Parent Versions.
