# Supplementary material for: Paving the Way for Outdoor Play: Examining Socio-Environmental Barriers to Community-Based Outdoor Play
Source: Int J Environ Res Public Health. 2021 Mar 31;18(7):3617. doi: 10.3390/ijerph18073617 (PMC8037806; doi:10.3390/ijerph18073617)

Graph of Estimated Marginal Means for Variable Set 1: Demographic and Household Characteristics

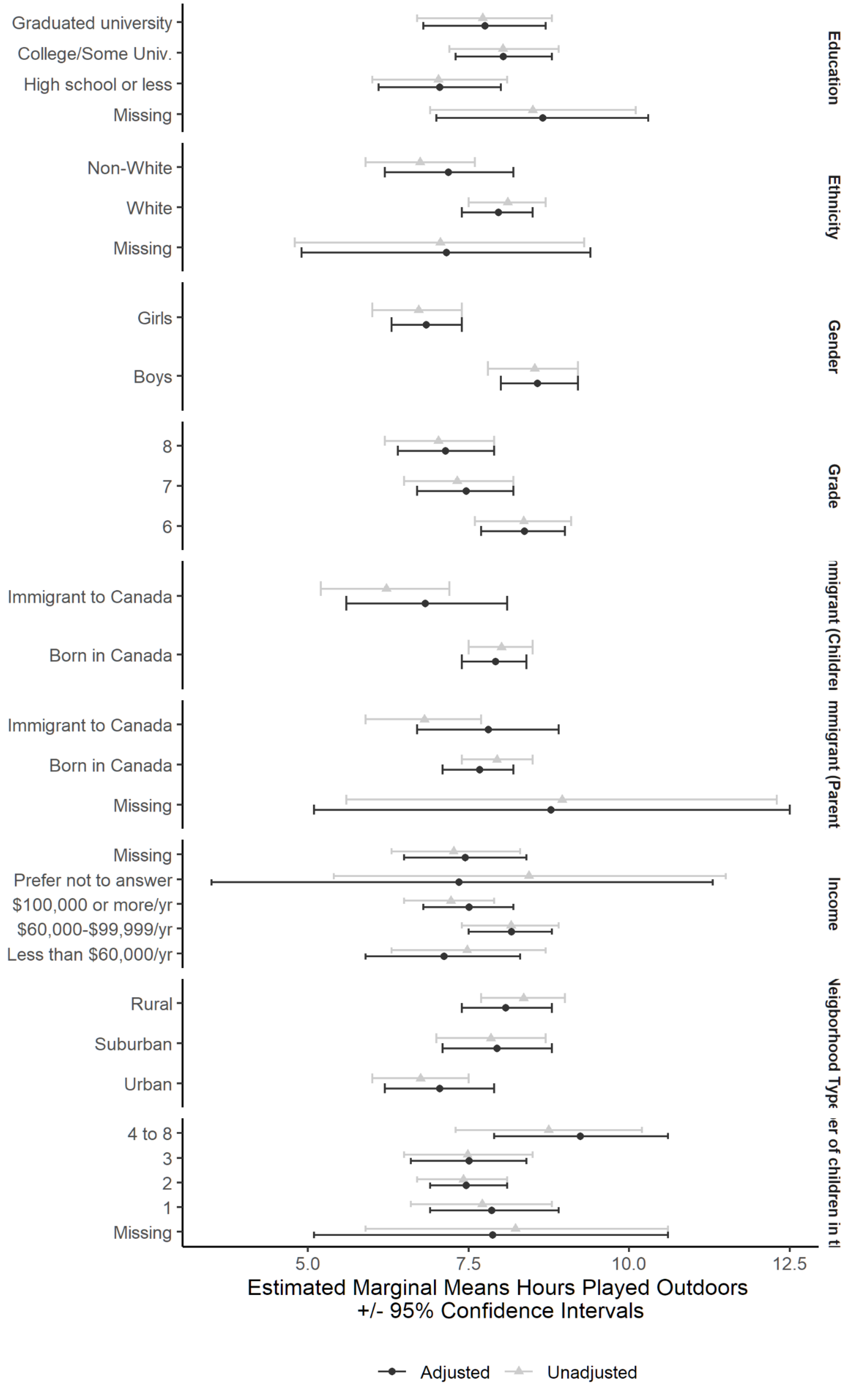

Graphs of Estimated Marginal Means for Variable Set 2: Time Available for Outdoor Free Play

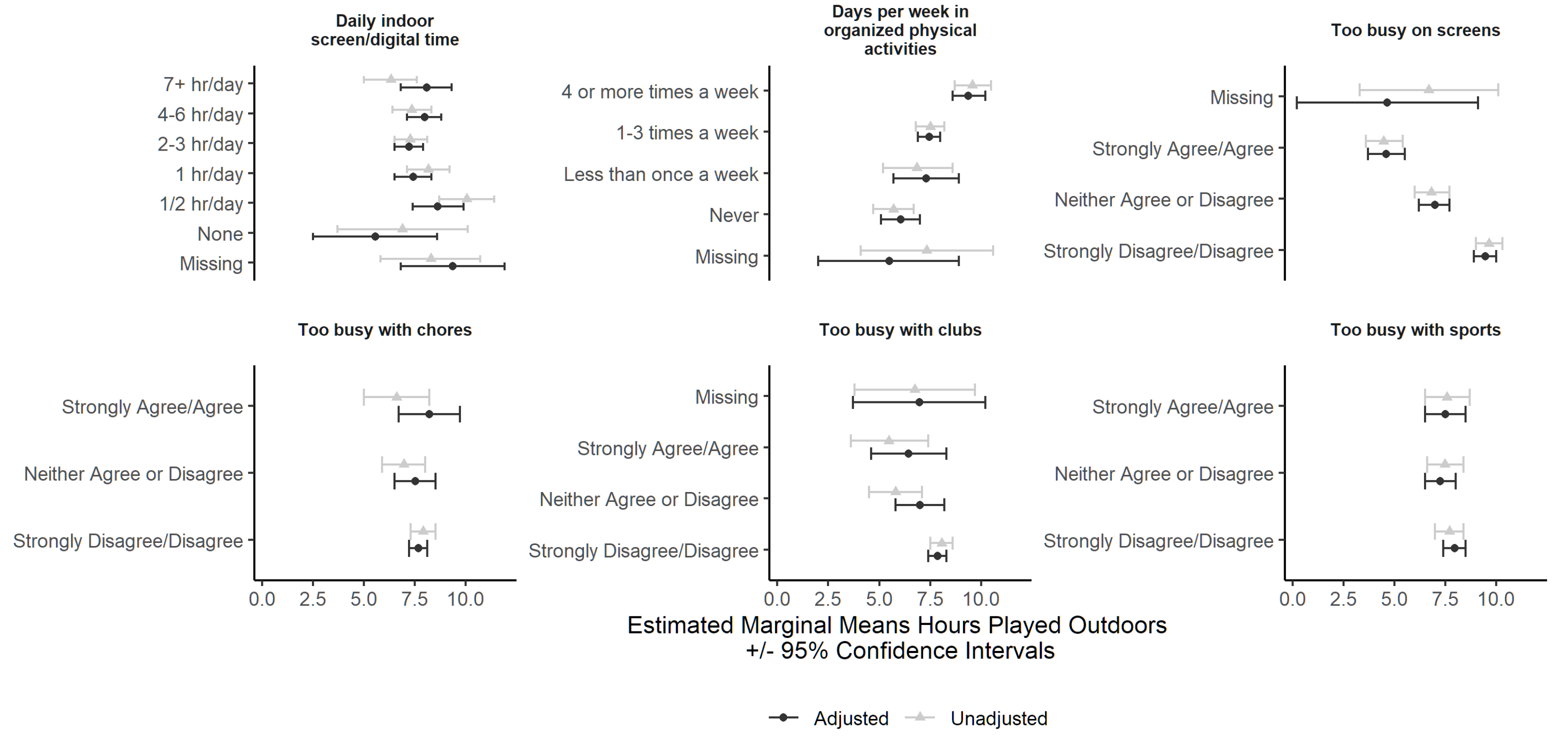

Graphs of Estimated Marginal Means for Variable Set 3: Children's License for Community Play and Mobility

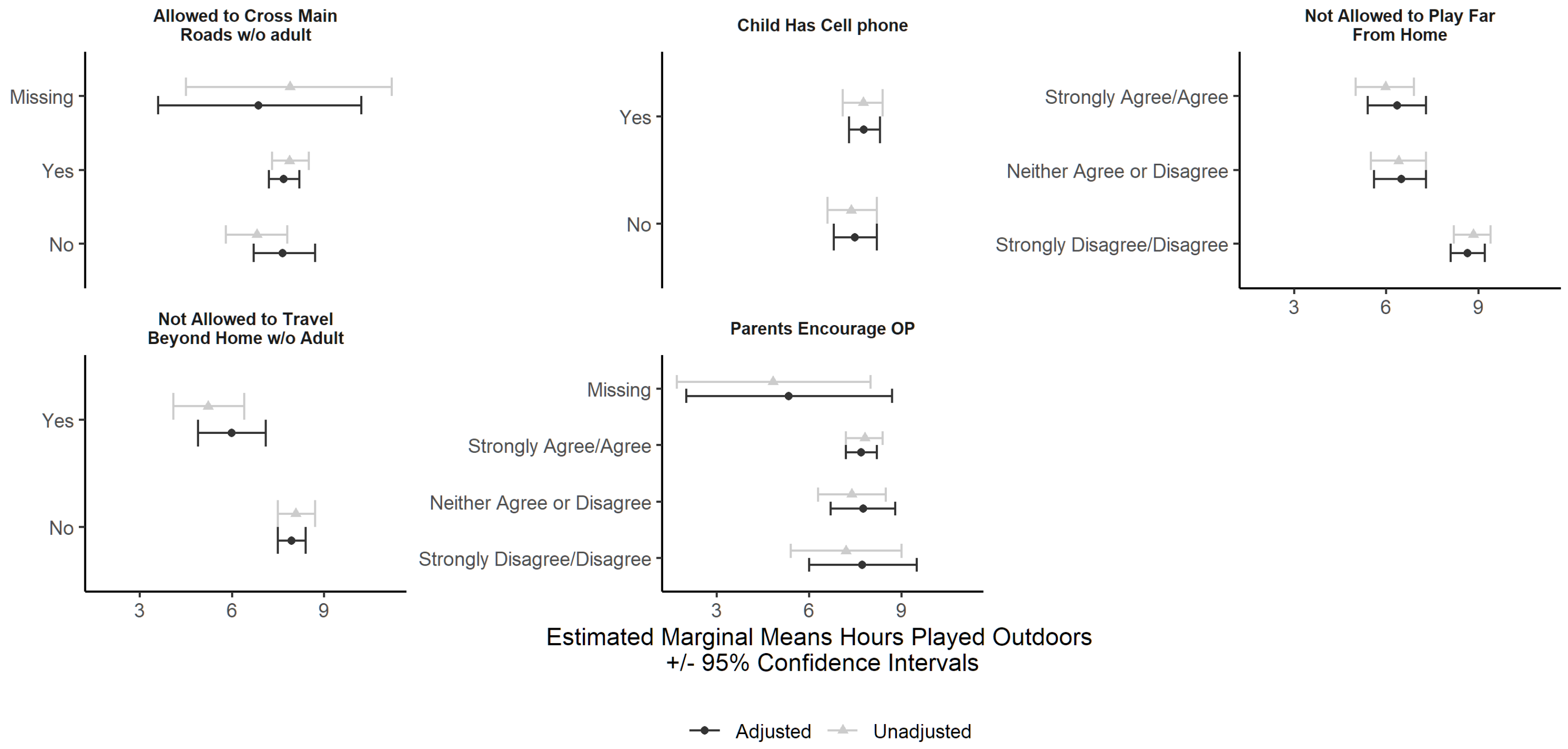

Graphs of Estimated Marginal Means for Variable Set 4: Socio-environmental Supports for Outdoor Play

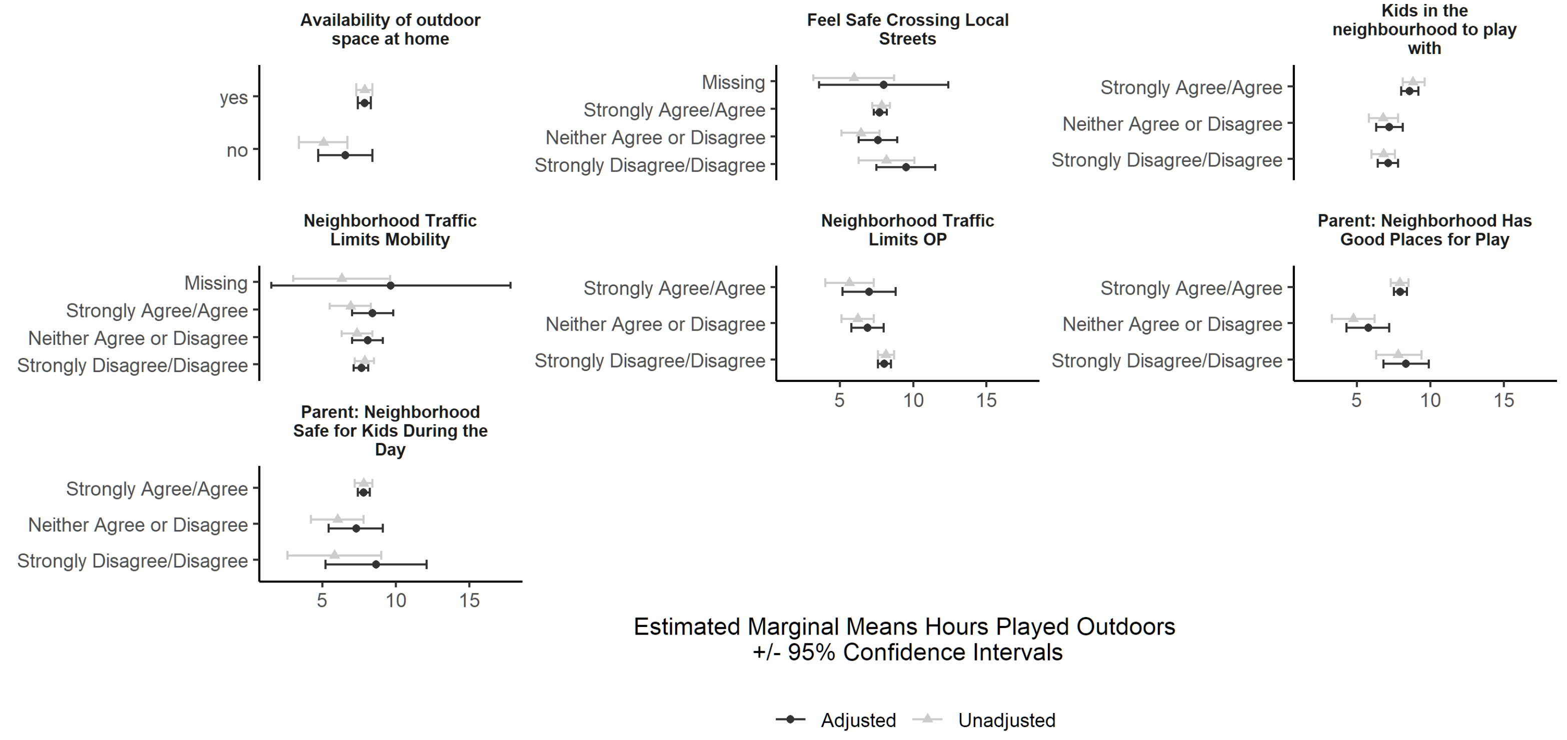

Graphs of Estimated Marginal Means for Variable Set 5: Child and Parent Attitudes Towards Outdoor Play

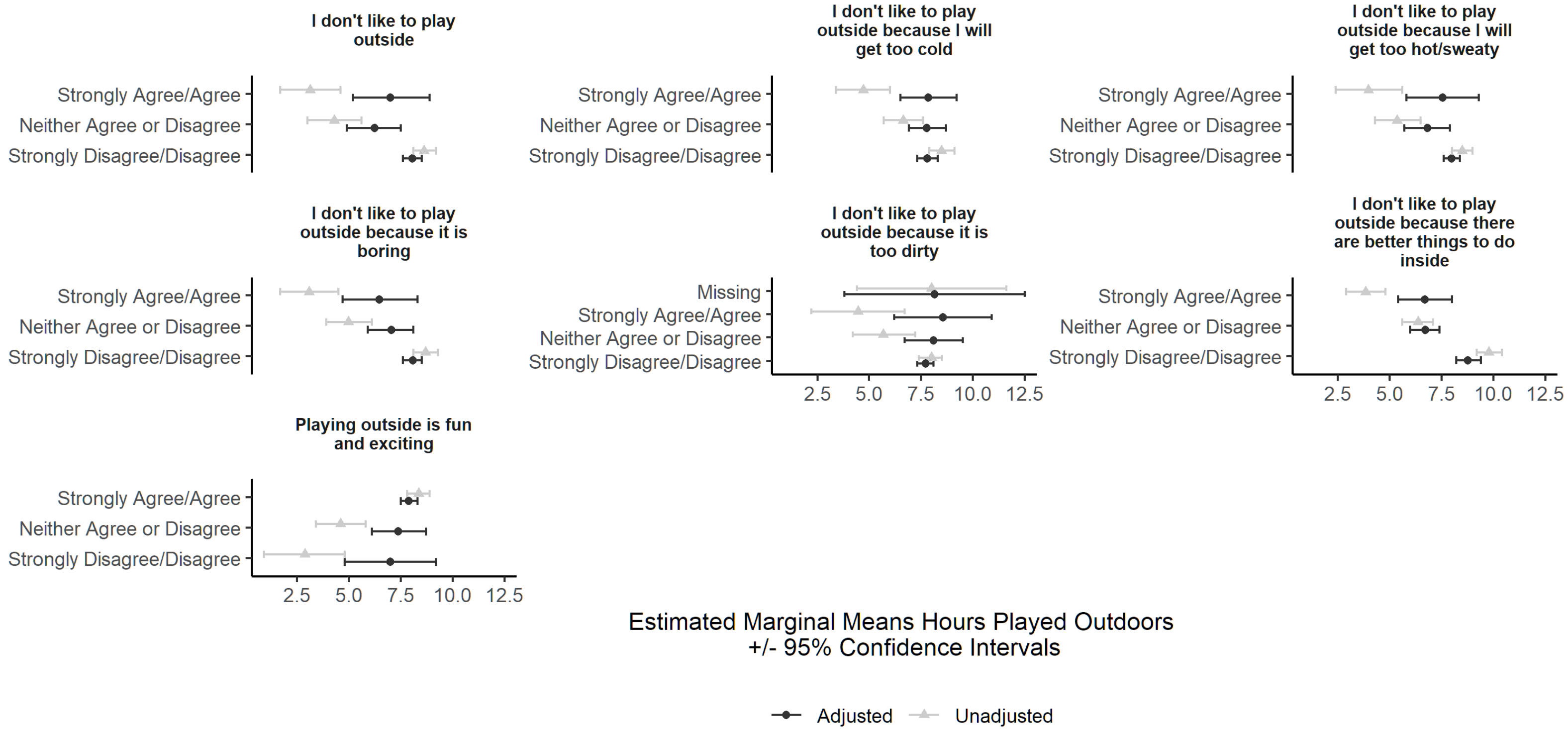

Supplement: Supplementary file 1 [file ijerph-18-03617-s001.zip › Updated Supplementary Mtl - Loebach et al/Graphs of Estimated Marginal Means.pdf]
