# Supplementary material for: Paving the Way for Outdoor Play: Examining Socio-Environmental Barriers to Community-Based Outdoor Play
Source: Int J Environ Res Public Health. 2021 Mar 31;18(7):3617. doi: 10.3390/ijerph18073617 (PMC8037806; doi:10.3390/ijerph18073617)
Supplement: Supplementary file 1 [file ijerph-18-03617-s001.zip › Updated Supplementary Mtl - Loebach et al/OutdoorPlayStudy_ParentSurvey.pdf]

Parent ID# |____|____|____|

School ID# |____|____|

PARENT QUESTIONNAIRE

Thank you for agreeing to join our Children's Outdoor Play Study! In this questionnaire, you will be asked questions about your child's play outdoors as well as questions about your attitudes, feelings and behaviors. We will also ask you questions about your activities, perceptions of your neighborhood environment and your physical health and mental well-being.

Before completing the questionnaire you should know that:

- The questionnaire will take about 20 minutes to complete.
- When answering questions about your child, answer for your child who is in Grade 6, 7 or 8. **If you have more than one child in Grades 6, 7 or 8,** please respond regarding your eldest child in those grades.
- You can skip any questions that you do not wish to answer.
- There are no right or wrong answers.
- Whether or not you complete the questionnaire will not affect your child's academic standing at her/his school.
- Your responses will be completely confidential. Your name will not appear anywhere on the questionnaire. The questionnaire has a number code that the researchers will use to connect your answers to your child's answers. Your child will be completing a separate questionnaire.
- When study results are reported, findings will be presented in a way that it will not be possible to identify any of your responses to the questions you are asked.

Please complete this survey using a BLACK PEN,
and be sure to fill in all circles completely. Thank you!

Children's Outdoor Play: Primary Caregiver Survey CAMH © 2017

Child's Primary Home Postal Code ________________ *(Optional)*

1. What is your gender? O Male O Female O Other (Please specify)_______

2. What is your year of birth? |____|____|____|____|

Thinking about your child in Grade 6, 7 or 8…

3. Not including recess, about how many days in an average week in the **Spring, Summer and Fall** does your child spend doing activities outside **without adult supervision**?

   O 0 none O 1 day O 2 days O 3 days O 4 days

   O 5 days O 6 days O 7 days O My child doesn't play outside without an adult

4. On days your child goes outside to play **without adult supervision** in the **Spring, Summer and Fall**, how much time does he/she typically play (excluding recess)?

   O <30 minutes O 2 hours

   O 30 minutes to <1 hour O 3 or more hours

   O 1 hour O My child doesn't play outside without an adult

5. Not including recess, about how many days in an average week during the **Winter** does your child play outside **without adult supervision**?

   O 0 none O 1 day O 2 days O 3 days O 4 days

   O 5 days O 6 days O 7 days O My child doesn't play outside without an adult

6. On days your child goes outside to play **without adult supervision** during the **Winter**, how much time does he/she typically play (excluding recess)?

   O <30 minutes O 2 hours

   O 30 minutes to <1 hour O 3 or more hours

   O 1 hour O My child doesn't play outside without an adult

7. Since the beginning of the school year, how often has your child played organized sports or taken lessons with a coach or instructor (swimming lessons, snowboarding, baseball, hockey, karate, dance, gymnastics etc.) not including school recess?

   O Never O 1-3 times a week

   O Less than once a week O 4 or more times a week

8. Does your child have a physical chronic health condition that limits their daily activities at home, school, or in the community?

   O A lot O Quite a bit O A little O Not at all

   O My child has no physical chronic health conditions

9. Please indicate how strongly you disagree or agree with the following statements.

		Strongly disagree	Somewhat disagree	Neither agree nor disagree	Somewhat agree	Strongly agree
a	Playing outside encourages too much aggressive behaviour	O	O	O	O	O
b	All playgrounds should contain natural elements	O	O	O	O	O
c	Playing outside would be good for my child's health	O	O	O	O	O
d	There is too much crime for my child to play outside	O	O	O	O	O
e	I think my child should go on nature hikes	O	O	O	O	O
f	Playing outside is a wasteful way for children to spend their free time	O	O	O	O	O
g	My neighbourhood is safe enough for children to play outside	O	O	O	O	O
h	My child gets too dirty when playing outside	O	O	O	O	O
i	I am afraid my child may be harmed by strangers outside	O	O	O	O	O
j	I worry that my child will be hurt by gangs if he/she plays outside	O	O	O	O	O
k	My child's learning can be stimulated by outdoor play	O	O	O	O	O
l	Playing outside hurts my child's school grades	O	O	O	O	O
m	Playing outdoors is a good way to improve hand-eye coordination	O	O	O	O	O

		Strongly disagree	Somewhat disagree	Neither agree nor disagree	Somewhat agree	Strongly agree
n	Other children in my neighbourhood are safe for my child to play around	O	O	O	O	O
o	Children who play outdoors gain confidence	O	O	O	O	O
p	I allow my child to have a wide range of recreational outdoor activities from which to choose	O	O	O	O	O
q	I feel that outdoor play interferes too much with my child's homework time	O	O	O	O	O
r	My child is easier to manage after spending time outside	O	O	O	O	O
s	I am afraid my child may be abducted outdoors	O	O	O	O	O
t	Taking part in outdoor recreation improves my child's communication skills	O	O	O	O	O
u	Outdoor activities over stimulate my child	O	O	O	O	O
v	Taking part in outdoor activities helps to build up my child's level of independence	O	O	O	O	O
w	Outdoor activities are a good way for my child to make friends	O	O	O	O	O
x	Participating in outdoor play is a good way for my child to get exercise	O	O	O	O	O
y	My child likes to play outside	O	O	O	O	O

10. Does your child use a cellphone?

O Yes O No

11. Do you have your child take a cellphone with them when they play outside **without an adult**?

O Never O Rarely O Sometimes O Most of the time

O Always O My child doesn't use a cell phone O My child doesn't play outside without an adult

12. Please answer each of the items regarding your child's feelings and behaviour.

		Not at all	Hardly ever	Sometimes	Most of the time	All the time
a	My child is quiet when he/she is with a group of kids	O	O	O	O	O
b	My child is afraid to invite other kids to do things with him/her because they might say no	O	O	O	O	O
c	My child feels shy even with kids my child knows well	O	O	O	O	O
d	It's hard for my child to ask other children to do things with him/her	O	O	O	O	O

13. What kind of grades does your child usually get in his/her classes at school?

O Mostly A's / above 80% / level 4

O Mostly B's / 70 - 79% / level 3

O Mostly C's / 60 - 69% / level 2

O Mostly D's / 50 - 59% / level 1

O Mostly letter grades below D's / below 50% / below level 1

14. What is your child's weight? ________ pounds

15. What is your child's height? ________ feet and ________ inches

16. Please answer each of the following regarding your child.

		Not at all true	A little true	Somewhat true	Mostly true	Completely true
a	To my child having fun time is more important than a minor injury	O	O	O	O	O
b	My child 'gets into things'	O	O	O	O	O
c	My child will 'follow along' and do what other kids are doing even if these are things that s/he shouldn't do	O	O	O	O	O
d	Other children can talk my child into doing fun risky things	O	O	O	O	O

		Not at all true	A little true	Somewhat true	Mostly true	Completely true
e	My child will go along with other kids and do something that could lead to injury	O	O	O	O	O
f	My child is a thrill seeker	O	O	O	O	O
g	I've never been surprised by the risks my child will take	O	O	O	O	O
h	My child will 'go along with the crowd' and do things that might lead to injury	O	O	O	O	O
i	My child likes taking risks even if these can lead to injury	O	O	O	O	O

17. For each statement, please check the answer that best describes you.

		Not at all true	A little true	Somewhat true	Mostly true	Completely true
a	I don't let my child out of my sight for too long	O	O	O	O	O
b	When my child is with certain friends, I monitor him/her more closely than when s/he is with other friends	O	O	O	O	O
c	I can leave my child alone for long periods of time without worrying something will happen	O	O	O	O	O
d	To keep my child safe, I need to be closely supervising at all times	O	O	O	O	O
e	I think that at his/her age, my child is quite capable of keeping him/herself safe from injury	O	O	O	O	O
f	As long as I generally know where my child is and what s/he is doing, I don't have to go check on him/her	O	O	O	O	O
g	When we are at home, it works out fine for me to just wait until my child calls or comes to get me if s/he needs help	O	O	O	O	O
h	I sometimes snoop around my child's room or belongings to be sure I haven't missed something I should be aware of	O	O	O	O	O

		Not at all true	A little true	Somewhat true	Mostly true	Completely true
i	I think it is important to monitor children closely even at these ages	O	O	O	O	O
j	By 6-10 years of age, I think that only children who are poor at following rules need constant supervision	O	O	O	O	O
k	I always check to see what my child is doing when s/he is out of sight for long	O	O	O	O	O
l	I usually only allow my child to play with friends when an adult is around to supervise closely	O	O	O	O	O
m	I think it is best to keep a child busy than let them have too much free time on their hands	O	O	O	O	O
n	I believe my child will follow the rules even if I am not there watching him/her	O	O	O	O	O
o	One way or another, I always figure out what my child is up to	O	O	O	O	O

18. Please indicate how strongly you agree or disagree with the following statements about your neighbourhood:

		Strongly disagree	Disagree	Neither agree nor disagree	Agree	Strongly agree
a	It is safe to walk alone in this neighbourhood after dark	O	O	O	O	O
b	It is safe for children to play outside during the day	O	O	O	O	O
c	There are good parks, playgrounds and play spaces in this neighbourhood	O	O	O	O	O
d	If there is a problem around here, the neighbours get together to deal with it	O	O	O	O	O
e	There are adults in the neighbourhood that children can look up to	O	O	O	O	O
f	People around here are willing to help their neighbours	O	O	O	O	O

		Strongly disagree	Disagree	Neither agree nor disagree	Agree	Strongly agree
g	You can count on adults in this neighbourhood to watch out that children are safe and don't get in trouble	O	O	O	O	O
h	When I'm away from home, I know that my neighbours will keep their eyes open for possible trouble	O	O	O	O	O

19. Is there anything else you would like to add about your child playing outside?

____

____

____

Now some questions about you…

20. How would you best describe yourself? (*Check all that apply*)

O Aboriginal (e.g., First Nations, Métis, Inuit)

O Black/African/Caribbean

O East Asian (e.g., Chinese, Japanese, Korean)

O Latin American/Hispanic (e.g., Mexican, South American, Central American)

O Middle Eastern (e.g., Egyptian, Iranian, Lebanese)

O South Asian (e.g., Indian, Pakistani, Sri Lankan)

O White/Caucasian

O Other [*Please specify]* ___________________________

21. How long have you lived in Canada?

O 2 years or less

O 3 – 5 years

O 6 – 10 years

O More than 10 years but was not born in Canada

O Born in Canada

22. How long have you lived at your current address?

O Less than one year

O 1 – 2 years

O 3 – 5 years

O 6-10 years

O More than 10 years

23. What best describes your main activities in the <u>past 12 months</u> (Check all that apply)?

O Working for pay

O Self-employed

O Going to school

O Caring for family

O Long-term illness/on disability

O Retired

O Unemployed/EI

O Unemployed/Ontario Works

O Other (specify) ____________________

24. What is the highest level of education you have completed?

- O Did not complete high school
- O Graduated high school
- O Completed some college
- O College diploma
- O Completed some university
- O University degree

25. How many children under 18 do you have living with you? (include biological, adopted, step-children, your partner's children or grandchildren)

|____|____| # children

26. Please estimate in which of the following groups your household income falls?

- O Less than $20,000
- O Between $20,000 and $29,999.99
- O Between $30,000 and $39,999.99
- O Between $40,000 and $49,999.99
- O Between $50,000 and $59,999.99
- O Between $60,000 and $69,999.99
- O Between $70,000 and $79,999.99
- O Between $80,000 and $89,999.99
- O Between $90,000 and $99,999.99
- O $100,000 or more
- O Prefer not to answer
- O I don't know

27. How often have you felt or behaved this way during the past week?

		**Rarely or none of the time**	**Some or little of the time**	**Occasionally or moderate amount of time**	**Most or all of the time**
a	I did not feel like eating; my appetite was poor	O	O	O	O
b	I felt that I could not shake off the blues or sadness even with help from my family or friends	O	O	O	O
c	I had trouble keeping my mind on what I was doing	O	O	O	O
d	I felt depressed	O	O	O	O
e	I felt that everything I did was an effort	O	O	O	O
f	I felt hopeful about the future	O	O	O	O
g	My sleep was restless/I did not sleep well	O	O	O	O
h	I was happy	O	O	O	O
i	I felt lonely	O	O	O	O

j	I enjoyed life	O	O	O	O
k	I had crying spells	O	O	O	O
l	I felt that people disliked me	O	O	O	O

28. About how many days in an <u>average week</u>, in the **Spring, Summer and Fall**, do you spend doing activities outside (e.g., playing with children, gardening, going for a walk, playing sports or doing exercise)?

    O 0 none   O 1 day   O 2 days   O 3 days

    O 4 days   O 5 days   O 6 days   O 7 days

29. On the days when you do activities outside in the **Spring, Summer and Fall,** how much time do you usually spend?

    O <30 minutes   O 1 hour   O 3 or more hours

    O 30 minutes to <1 hour   O 2 hours

30. About how many days in an <u>average week</u>, in the **Winter**, do you spend doing activities outside?

    O 0 none   O 1 day   O 2 days   O 3 days

    O 4 days   O 5 days   O 6 days   O 7 days

31. On the days when you do activities outside in the **Winter,** how much time do you usually spend?

    O <30 minutes   O 1 hour   O 3 or more hours

    O 30 minutes to <1 hour   O 2 hours

32. In a <u>typical week</u>, do you spent 150 or more minutes on physical activity that makes you breathe harder or sweat?

    O Yes   O No   O Don’t know

33. How would you describe your health?

    O Excellent   O Good   O Fair   O Poor

Thank you!
