# Supplementary material for: Paving the Way for Outdoor Play: Examining Socio-Environmental Barriers to Community-Based Outdoor Play
Source: Int J Environ Res Public Health. 2021 Mar 31;18(7):3617. doi: 10.3390/ijerph18073617 (PMC8037806; doi:10.3390/ijerph18073617)
Supplement: Supplementary file 1 [file ijerph-18-03617-s001.zip › Updated Supplementary Mtl - Loebach et al/OutdoorPlayStudy_YouthSurvey.pdf]

Youth ID# |____|____|____|

School ID# |____|____|

YOUTH QUESTIONNAIRE

Thank you for agreeing to join our Outdoor Play Study! In this questionnaire, you will be asked questions about your activities, attitudes and feelings, physical health and mental well-being.

Before completing the questionnaire you should know that:

- The questionnaire will take about 20 minutes to complete.
- You can skip any questions that you do not wish to answer.
- There are no right or wrong answers.
- Whether or not you complete the questionnaire will not affect your academic standing at this school.
- Your responses will be completely confidential (private). Your name will not appear anywhere on the questionnaire.
- When study results are reported, findings will be presented in such a way that it will not be possible to identify any of your responses to the questions you are asked.

Please complete this survey using a BLACK PEN,
and be sure to fill in all circles completely. Thank you!

Home postal code ___________________ *(Optional)*

1. What grade are you in? ⑥ ⑦ ⑧

2. What is your gender? O Male O Female O Other (*Please specify*)______________

3. How would you best describe yourself? (*Check all that apply*)

   O Aboriginal (e.g., First Nation, Métis, Inuit)

   O Black/African/Caribbean

   O East Asian (e.g., Chinese, Japanese, Korean)

   O Latin American/Hispanic (e.g., Mexican, South American, Central American)

   O Middle Eastern (e.g., Egyptian, Iranian, Lebanese)

   O South Asian (e.g., Indian, Pakistani, Sri Lankan)

   O White/Caucasian

   O Other (*Please specify*) ___________________________

4. How long have you lived in Canada?

   O 2 years or less O 3 – 5 years O 6 – 10 years

   O More than 10 years O Born in Canada

5. Does your home have a yard or outdoor space where you can run and play games or just have fun by yourself?

   O Yes O No

6. Not including recess, about how many days in a <u>week</u> do you usually play outside **<u>during the Spring, Summer and Fall</u>**?

   ⓪ ① ② ③ ④ ⑤ ⑥ ⑦

7. On days you play outside (excluding recess) **<u>during the Spring, Summer and Fall</u>**, about how much time do you usually play?

   O Less than 30 minutes O 1 hour O 3 or more hours

   O 30 minutes to less than 1 hour O 2 hours

8. Not including recess, about how many days in a <u>week</u> do you usually play outside **<u>during the Winter</u>?**

   ⓪ ① ② ③ ④ ⑤ ⑥ ⑦

9. On days you play outside (excluding recess) **during the Winter**, about how much time do you usually play?

   O Less than 30 minutes O 1 hour O 3 or more hours

   O 30 minutes to less than 1 hour O 2 hours

10. Not including recess, when you play outside, who are you usually with? (*Check all that apply*)

    O A parent/guardian

    O Pet(s)

    O I play alone

    O I play with my brother(s) or sister(s)

    O I play with my friend(s)

    O Someone else [Who?]: ______________________________

11. Not including recess, about how many days in a week do you usually play outside **without an adult playing with you or watching you**?

    O 0 none O 1 day O 2 days O 3 days O 4 days

    O 5 days O 6 days O 7 days O I don't play outside without an adult

12. Not including recess, on days you play outside **without an adult playing with you or watching you**, how much time do you usually play?

    O Less than 30 minutes O 2 hours

    O 30 minutes to less than1 hour O 3 or more hours

    O 1 hour O I don't play outside without an adult

13. Not counting travelling to or from school, how far from your home do you travel by yourself or with friends, **without an adult**?

    O I don't travel away from home without an adult/I stay in my yard

    ______ By walking: number of minutes from home one way

    ______ By biking: number of minutes from home one way OR O I don't have a bike

    ______ By public transit: number of minutes from home one way OR O I don't use public transit

14. Are you allowed to cross main roads without an adult?

    O Yes O No

15. When you go outside to play **without an adult,** where do you usually go? (*Please check all that apply*)

- O I'm not allowed to go outside to play without an adult
- O Driveway, dead end or shared neighbourhood space
- O Parking lot
- O Open field
- O Your own yard
- O Playground
- O Sports field
- O Parks
- O Street or roadway
- O Wooded area or wetland
- O Pond, creek, river or lake
- O A friend's backyard
- O School or church yard
- O Other places (Please specify)____________________

16. The next questions ask you about your experiences and feelings about playing outdoors. How much do you agree with each statement?

		**Strongly disagree**	**Disagree**	**Neither agree nor disagree**	**Agree**	**Strongly agree**
a	Playing outside helps me think more clearly	O	O	O	O	O
b	Playing outside makes me healthier	O	O	O	O	O
c	When I'm angry, playing outside calms me down	O	O	O	O	O
d	I learn new things when I play outside	O	O	O	O	O
e	I feel like I have freedom when I play outside	O	O	O	O	O
f	I like to make up games when I'm outside	O	O	O	O	O
g	I like to explore new places outside	O	O	O	O	O
h	I am afraid of getting lost outside	O	O	O	O	O
i	I don't like playing outside because there are strangers	O	O	O	O	O

		Strongly disagree	Disagree	Neither agree nor disagree	Agree	Strongly agree
j	I am afraid of wild animals or insects outside	O	O	O	O	O
k	I am afraid of getting hurt if I play outside	O	O	O	O	O
l	I don't like playing outside because there are people drinking alcohol or with drugs	O	O	O	O	O
m	Playing outside gives me a chance to make new friends	O	O	O	O	O
n	I am afraid someone will bully me when I play outside	O	O	O	O	O
o	I don't like playing outside because the streets are busy with traffic (e.g., cars, trucks, etc.)	O	O	O	O	O
p	Playing outside is fun and exciting	O	O	O	O	O
q	Playing outside allows me to do things on my own	O	O	O	O	O
r	I don't like to play outside	O	O	O	O	O
s	I don't like to play outside because it is boring	O	O	O	O	O
t	There are a lot of kids in my neighbourhood to play with outside	O	O	O	O	O
u	I don't like to play outside because It is too dirty	O	O	O	O	O
v	I don't like to play outside because I will get too hot/sweaty	O	O	O	O	O
w	I don't like to play outside because I will get too cold	O	O	O	O	O
x	I don't like to play outside because there are better things to do inside	O	O	O	O	O

		Strongly disagree	Disagree	Neither agree nor disagree	Agree	Strongly agree
y	I like to play outside in the summer	O	O	O	O	O
z	I like to play outside in the winter	O	O	O	O	O
aa	My parents/caregivers encourage me to play outside	O	O	O	O	O

17. Please indicate how strongly you agree or disagree with the following statements:

		Strongly disagree	Disagree	Neither Agree nor Disagree	Agree	Strongly agree
a	I'm not allowed to go outside	O	O	O	O	O
b	I'm not allowed to play far from home	O	O	O	O	O
c	I'm only allowed to play outside with an adult	O	O	O	O	O
d	People treat me unfairly because of my race/ethnicity when I play outside	O	O	O	O	O
e	I'm too busy with organized sports (e.g. hockey, gymnastics) for other play outside	O	O	O	O	O
f	I'm too busy with clubs (e.g. Drama, Guides/Scouts etc.) to play outside	O	O	O	O	O
g	I'm too busy with chores to play outside (e.g., babysitting, cleaning)	O	O	O	O	O
h	I'm too busy playing video games, watching TV, on the internet, social media or texting to play outside	O	O	O	O	O
i	I'm too busy with other things to play outside (*What other things?* ________________________)	O	O	O	O	O

18. Do you have a cellphone?

O Yes O No

19. Does your parent(s)/guardian(s) have you take a cellphone with you when you play outside **<u>without an adult</u>**?

O Never O Rarely O Sometimes O Most of the time
O Always O I don't have a cellphone

20. When you play outside, do you text, use social media, or play games on your cell phone?

O Never O Rarely O Sometimes O Most of the time
O Always O I don't have a cellphone

21. When you play outside without an adult, how often do you do the following things?

		**I don't play outside without an adult**	**Never**	**Rarely**	**Some times**	**Often**	**Very often**
a	Play rough and tumble games (e.g. wrestling, playfighting)	O	O	O	O	O	O
b	Ride a bicycle, skateboard, ski at high speed	O	O	O	O	O	O
c	Climb a cliff, building, tall tree or fence	O	O	O	O	O	O
d	Play near or around a deep or icy river, pond, or lake	O	O	O	O	O	O
e	Jump or swing from a high place	O	O	O	O	O	O
f	Play with sharp objects or tools (e.g. knives, saws, axes)	O	O	O	O	O	O
g	Go exploring where you could get lost	O	O	O	O	O	O

22. <u>Over the past 7 days</u>, on how many days were you physically active for a total of at least 60 minutes per day (add up all the time you spent on any kind of physical activity that increased your heart rate or made you breathe hard some of the time)—examples are brisk walking, running, jogging, weight lifting, roller blading, biking, dancing, skateboarding, swimming, soccer, hockey, basketball, football, tennis.

⓪ ① ② ③ ④ ⑤ ⑥ ⑦

23. How many hours a week do you usually exercise or do any activity in your free time so much that you get out of breath or sweat?

O None
O ½ hour a week
O 1 hour a week
O 2-3 hours a week
O 4-6 hours a week
O 7 or more hours a week

24. Since the beginning of the school year, how often have you played organized sports or taken lessons with a coach or instructor (swimming lessons, snowboarding, baseball, hockey, karate, dance, gymnastics etc.) not including school recess?

O Never
O 1-3 times a week
O Less than once a week
O 4 or more times a week

25. In the last 7 days, about how many hours a day, did you spend indoors watching TV/movies, playing video/computer games, on a computer or phone chatting, texting, e-mailing, or surfing the internet?

O None
O ½ hour a day
O 1 hour a day
O 2-3 hours a day
O 4-6 hours a day
O 7 or more hours a day

26. How strongly do you agree or disagree with the following statements about your local neighbourhood?

		**Strongly disagree**	**Disagree**	**Neither Agree nor Disagree**	**Agree**	**Strongly agree**
a	The traffic makes it difficult or unpleasant for me to walk	O	O	O	O	O
b	The speed of traffic on most streets is usually slow (50 kph or less)	O	O	O	O	O
c	Most drivers go faster than the posted speed limits	O	O	O	O	O
d	I'm afraid of being taken or hurt by a stranger in a local park	O	O	O	O	O
e	Streets have good lighting at night	O	O	O	O	O
f	Walkers and bikers can be easily seen by people in their homes	O	O	O	O	O
g	There are crosswalks and signals on busy streets	O	O	O	O	O
h	There is a high crime rate	O	O	O	O	O

		Strongly disagree	Disagree	Neither Agree nor Disagree	Agree	Strongly agree
i	I'm afraid of being taken or hurt by a stranger on local streets	O	O	O	O	O
j	I'm afraid of being taken or hurt by a stranger in my yard, driveway or apartment common area	O	O	O	O	O
k	I'm afraid of being taken or hurt by a known "bad" person in my neighbourhood	O	O	O	O	O
l	I feel safe crossing the streets in my neighbourhood	O	O	O	O	O

27. Below is a list of statements that describe some of the feelings and behaviours of young people. For each statement, please select the response that best describes you now or within the past 6 months.

		Never or not true	Sometimes or somewhat true	Often or very true
a	I am nervous and tense	O	O	O
b	I am too fearful or anxious	O	O	O
c	I am anxious or on edge	O	O	O
d	I find it hard to stop worrying	O	O	O

28. In the last 4 weeks did you feel that you were under any stress, strain, or pressure?

O Yes, almost more than I could take   O Yes, a little   O Yes, some

O Yes, a lot   O Not at all

29. How often have you felt or behaved this way during the past week (7 days)?

		Never or rarely	Some times	Often	Always
a	I did not feel like eating; my appetite was poor	O	O	O	O
b	I felt I could not shake off the blues or sadness even with help from my family or friends	O	O	O	O

		Never or rarely	Some times	Often	Always
c	I had trouble keeping my mind on what I was doing	O	O	O	O
d	I felt depressed	O	O	O	O
e	I felt like I was too tired to do things	O	O	O	O
f	I felt hopeful about the future	O	O	O	O
g	My sleep was restless/I did not sleep well	O	O	O	O
h	I was happy	O	O	O	O
i	I felt lonely	O	O	O	O
j	I enjoyed life	O	O	O	O
k	I had crying spells	O	O	O	O
l	I felt people disliked me	O	O	O	O

30. Please tell us how much each of the statements below describe you.

		Strongly disagree	Disagree	Neither agree nor disagree	Agree	Strongly agree
a	I can solve many of the problems I have on my own	O	O	O	O	O
b	I can change many of the important things in my life	O	O	O	O	O
c	I do well even when things are tough	O	O	O	O	O
d	What happens to me mostly depends on me	O	O	O	O	O
e	I can do just about anything I really set my mind to do	O	O	O	O	O

31. How would you describe your physical health?

O Excellent O Good O Fair O Poor

32. What kind of grades do you usually get in your classes at school?

- O Mostly A’s / above 80% / level 4
- O Mostly B’s / 70 - 79% / level 3
- O Mostly C’s / 60 - 69% / level 2
- O Mostly D’s / 50 - 59% / level 1
- O Mostly letter grades below D’s / below 50% / below level 1

Almost done! Just a few questions about your parents or guardians….

33. How many parents/guardians do you live with?

- O One parent/guardian
- O Two parents/guardians
- O Split between parents/guardians
- O Something else (Please specify): ________________________

34. What is the highest level of education your mother/female guardian completed?

- O No mother/female guardian
- O Did not complete high school
- O Graduated high school
- O Completed some college
- O College diploma
- O Completed some university
- O University degree
- O Don’t know

35. What is the highest level of education your father/male guardian completed?

- O No father/male guardian
- O Did not complete high school
- O Graduated high school
- O Completed some college
- O College diploma
- O Completed some university
- O University degree
- O Don’t know

Thank you!
