# Supplementary material for: Paving the Way for Outdoor Play: Examining Socio-Environmental Barriers to Community-Based Outdoor Play
Source: Int J Environ Res Public Health. 2021 Mar 31;18(7):3617. doi: 10.3390/ijerph18073617 (PMC8037806; doi:10.3390/ijerph18073617)
Supplement: Supplementary file 1 [file ijerph-18-03617-s001.zip › Updated Supplementary Mtl - Loebach et al/Spearman Correlation Matrices.docx]

**Spearman Correlation matrix for Model 2: Time Available for Outdoor Free Play**

|  | Too busy with sports | Too busy with clubs | Too busy with chores | Too busy on screens | Days per week in organized physical activities | Daily indoor screen/digital time |
| --- | --- | --- | --- | --- | --- | --- |
| Too busy with sports | 1 |  |  |  |  |  |
|  | 818 |  |  |  |  |  |
|  |  |  |  |  |  |  |
| Too busy with clubs | 0.1977* | 1 |  |  |  |  |
|  | 805 | 809 |  |  |  |  |
|  | <0.0001 |  |  |  |  |  |
| Too busy with chores | 0.2209* | 0.2343* | 1 |  |  |  |
|  | 814 | 806 | 819 |  |  |  |
|  | <0.0001 | <0.0001 |  |  |  |  |
| Too busy on screens | 0.0172 | 0.1243* | 0.1379* | 1 |  |  |
|  | 810 | 802 | 813 | 815 |  |  |
|  | 0.6243 | 0.0004 | 0.0001 |  |  |  |
| Days per week in organized physical activities | 0.4227* | -0.0179 | -0.0473 | -0.1984* | 1 |  |
|  | 803 | 794 | 805 | 801 | 811 |  |
|  | <0.0001 | 0.6152 | 0.1802 | <0.0001 |  |  |
| Daily indoor screen/digital time | -0.0114 | 0.0379 | 0.0292 | 0.4161* | -0.1554* | 1 |
|  | 792 | 784 | 795 | 792 | 794 | 800 |
|  | 0.7493 | 0.2898 | 0.4109 | <0.0001 | <0.0001 |  |

*indicates significant at alpha level 0.05.

Spearman correlation was used because most of the variables were ordinal.

**Spearman Correlation Matrix for Model 3: Children’s License for Community Activity & Mobility**

|  | Not Allowed to Travel Beyond Home w/o Adult | Not Allowed to Play Far From Home | Allowed to Cross Main Roads w/o Adult | Child has Cell Phone | Parents Encourage OP | Parent Attitude Towards Level of Supervision |
| --- | --- | --- | --- | --- | --- | --- |
| Not Allowed to Travel Beyond Home w/o Adult | 1 |  |  |  |  |  |
|  | 826 |  |  |  |  |  |
|  |  |  |  |  |  |  |
| Not Allowed to Play Far From Home | 0.2585* | 1 |  |  |  |  |
|  | 816 | 816 |  |  |  |  |
|  | <0.0001 |  |  |  |  |  |
| Allowed to Cross Main Roads w/o adult | -0.1992* | -0.3527* | 1 |  |  |  |
|  | 810 | 801 | 810 |  |  |  |
|  | <0.0001 | <0.0001 |  |  |  |  |
| Child Has Cell phone | -0.0423 | -0.1183* | 0.1831* | 1 |  |  |
|  | 817 | 809 | 801 | 817 |  |  |
|  | 0.2277 | 0.0007 | <0.0001 |  |  |  |
| Parents  Encourage OP | -0.1020* | -0.0709* | 0.0203 | -0.0396 | 1 |  |
|  | 810 | 803 | 794 | 802 | 810 |  |
|  | 0.0037 | 0.0445 | 0.5685 | 0.263 |  |  |
| Parent attitude towards level of children supervision (scale 0 -100) | 0.1705* | 0.2154* | -0.1804* | -0.0022 | -0.0203 | 1 |
|  | 814 | 804 | 798 | 805 | 798 | 814 |
|  | <0.0001 | <0.0001 | <0.0001 | 0.9507 | 0.5678 |  |

**Spearman Correlation Matrix for Model 4: Socio-environmental Supports for Outdoor Play**

|  | Availability of outdoor space at home | Nbhd Traffic Limits OP | Kids in the nbhd to play with | Nbhd Traffic Limits Mobility | Feel safe crossing local streets | Parent: Nbhd Safe for Kids -day | Parent: Nbhd has good places for play | Child perceived nbhd crime | Parent perception of nbhd social cohesion |
| --- | --- | --- | --- | --- | --- | --- | --- | --- | --- |
| Availability of outdoor space at home | 1 |  |  |  |  |  |  |  |  |
|  | 825 |  |  |  |  |  |  |  |  |
|  |  |  |  |  |  |  |  |  |  |
| Neighborhood Traffic Limits OP | -0.0895* | 1 |  |  |  |  |  |  |  |
|  | 821 | 822 |  |  |  |  |  |  |  |
|  | 0.0103 |  |  |  |  |  |  |  |  |
| Kids in the neighbourhood to play with | 0.0924* | -0.0719* | 1 |  |  |  |  |  |  |
|  | 817 | 816 | 818 |  |  |  |  |  |  |
|  | 0.0082 | 0.0399 |  |  |  |  |  |  |  |
| Neighborhood Traffic Limits Mobility | -0.0595 | 0.3095* | -0.0849* | 1 |  |  |  |  |  |
|  | 811 | 808 | 804 | 812 |  |  |  |  |  |
|  | 0.0905 | <0.0001 | 0.0161 |  |  |  |  |  |  |
| Feel Safe  Crossing Local Streets | 0.1339* | -0.2950* | 0.2049* | -0.2432* | 1 |  |  |  |  |
|  | 806 | 804 | 801 | 801 | 807 |  |  |  |  |
|  | 0.0001 | <0.0001 | <0.0001 | <0.0001 |  |  |  |  |  |
| Parent: Neighborhood Safe for Kids During the Day | 0.1996* | -0.1627* | 0.0855* | -0.1044* | 0.0861* | 1 |  |  |  |
|  | 819 | 816 | 812 | 807 | 801 | 820 |  |  |  |
|  | <0.0001 | <0.0001 | 0.0148 | 0.003 | 0.0148 |  |  |  |  |
| Parent: Neighborhood Has Good Places for Play | 0.1224* | -0.1211* | 0.0759* | -0.0551 | 0.1123* | 0.2678* | 1 |  |  |
|  | 818 | 815 | 811 | 807 | 801 | 819 | 819 |  |  |
|  | 0.0005 | 0.0005 | 0.0306 | 0.1181 | 0.0015 | <0.0001 |  |  |  |
| Child Perceived Neighbourhood Crime | -0.1079* | 0.3600* | -0.0788* | 0.3601* | -0.2712* | -0.1397* | -0.0704* | 1 |  |
|  | 808 | 807 | 804 | 806 | 802 | 803 | 803 | 809 |  |
|  | 0.0021 | <0.0001 | 0.0254 | <0.0001 | <0.0001 | 0.0001 | 0.0461 |  |  |
| Parent Perception of Neighbourhood Cohesion | 0.2379* | -0.1027* | 0.1664* | -0.1074* | 0.1421* | 0.2686* | 0.2099* | -0.0956* | 1 |
|  | 817 | 814 | 810 | 805 | 799 | 818 | 817 | 801 | 818 |
|  | <0.0001 | 0.0033 | <0.0001 | 0.0023 | 0.0001 | <0.0001 | <0.0001 | 0.0068 |  |

**Spearman Correlation Matrix for Model 5: Child and Parent Attitudes Towards Outdoor Play**

|  | Playing outside is fun and exciting | I don’t like to play outside | I don’t like to play outside - boring | I don’t like to play outside – too dirty | I don’t like to play outside – get too hot | I don’t like to play outside – get too cold | I don’t like to play outside – better inside | Parent attitudes towards OP | Child perceived benefits of OP | Child perceived fears of OP |
| --- | --- | --- | --- | --- | --- | --- | --- | --- | --- | --- |
| Playing outside is fun and exciting | 1 |  |  |  |  |  |  |  |  |  |
|  | 823 |  |  |  |  |  |  |  |  |  |
|  |  |  |  |  |  |  |  |  |  |  |
| I don't like to play outside | -0.5348* | 1 |  |  |  |  |  |  |  |  |
|  | 817 | 819 |  |  |  |  |  |  |  |  |
|  | <0.0001 |  |  |  |  |  |  |  |  |  |
| I don't like to play outside because it is boring | -0.5042* | 0.6046* | 1 |  |  |  |  |  |  |  |
|  | 817 | 814 | 819 |  |  |  |  |  |  |  |
|  | <0.0001 | <0.0001 |  |  |  |  |  |  |  |  |
| I don't like to play outside because it is too dirty | -0.2488* | 0.2885* | 0.3168* | 1 |  |  |  |  |  |  |
|  | 814 | 810 | 811 | 815 |  |  |  |  |  |  |
|  | <0.0001 | <0.0001 | <0.0001 |  |  |  |  |  |  |  |
| I don't like to play outside because I will get too hot/sweaty | -0.1747* | 0.2201* | 0.3193* | 0.3636* | 1 |  |  |  |  |  |
|  | 817 | 814 | 814 | 812 | 818 |  |  |  |  |  |
|  | <0.0001 | <0.0001 | <0.0001 | <0.0001 |  |  |  |  |  |  |
| I don't like to play outside because I will get too cold | -0.1683* | 0.2152* | 0.2528* | 0.2814* | 0.4305* | 1 |  |  |  |  |
|  | 820 | 815 | 816 | 814 | 817 | 821 |  |  |  |  |
|  | <0.0001 | <0.0001 | <0.0001 | <0.0001 | <0.0001 |  |  |  |  |  |
| I don't like to play outside because there are better things to do inside | -0.4413* | 0.5009* | 0.5289* | 0.2886* | 0.3672* | 0.3427* | 1 |  |  |  |
|  | 819 | 815 | 815 | 812 | 816 | 819 | 820 |  |  |  |
|  | <0.0001 | <0.0001 | <0.0001 | <0.0001 | <0.0001 | <0.0001 |  |  |  |  |
| Parent attitudes towards playing outdoors (PACOR) | 0.1427* | -0.0805* | -0.1464* | -0.1068* | -0.2066* | -0.1723* | -0.2168* | 1 |  |  |
|  | 819 | 815 | 815 | 811 | 814 | 817 | 816 | 822 |  |  |
|  | <0.0001 | 0.0215 | <0.0001 | 0.0023 | <0.0001 | <0.0001 | <0.0001 |  |  |  |
| Child Perceived Benefits of Outdoor Play (ATOP) | 0.4508* | -0.3897* | -0.3566* | -0.1350* | -0.2035* | -0.2270* | -0.3877* | 0.2318* | 1 |  |
|  | 822 | 817 | 817 | 814 | 817 | 820 | 819 | 820 | 824 |  |
|  | <0.0001 | <0.0001 | <0.0001 | 0.0001 | <0.0001 | <0.0001 | <0.0001 | <0.0001 |  |  |
| Child Perceived Fears of Outdoor Play (ATO) | -0.0528 | 0.1387* | 0.1577* | 0.2923* | 0.3566* | 0.3702* | 0.2540* | -0.2138* | -0.0923* | 1 |
|  | 822 | 818 | 817 | 814 | 818 | 820 | 820 | 819 | 822 | 823 |
|  | 0.1304 | 0.0001 | <0.0001 | <0.0001 | <0.0001 | <0.0001 | <0.0001 | <0.0001 | 0.0081 |  |
